# Recombinant BCG Vaccines Reduce Pneumovirus-Caused Airway Pathology by Inducing Protective Humoral Immunity

**DOI:** 10.3389/fimmu.2018.02875

**Published:** 2018-12-06

**Authors:** Jorge A. Soto, Nicolás M. S. Gálvez, Claudia A. Rivera, Christian E. Palavecino, Pablo F. Céspedes, Emma Rey-Jurado, Susan M. Bueno, Alexis M. Kalergis

**Affiliations:** ^1^Departamento de Genética Moleculary Microbiología, Facultad de Ciencias Biológicas, Millennium Institute of Immunology and Immunotherapy, Pontificia Universidad Católica de Chile, Santiago, Chile; ^2^Departamento de Endocrinología, Facultad de Medicina, Pontificia Universidad Católica de Chile, Santiago, Chile

**Keywords:** hRSV, hMPV, antibodies, humoral immune response, vaccine, respiratory virus, BCG

## Abstract

The Human Respiratory Syncytial Virus (hRSV) and the Human Metapneumovirus (hMPV) are two pneumoviruses that are leading agents causing acute lower respiratory tract infections (ALRTIs) affecting young infants, the elderly, and immunocompromised patients worldwide. Since these pathogens were first discovered, many approaches for the licensing of safe and effective vaccines have been explored being unsuccessful to date. We have previously described that immunization with recombinant strains of *Mycobacterium bovis* Bacillus Calmette-Guérin (rBCG) expressing the hRSV nucleoprotein (rBCG-N) or the hMPV phosphoprotein (rBCG-P) induced immune protection against each respective virus. These vaccines efficiently promoted viral clearance without significant lung damage, mainly through the induction of a T helper 1 cellular immunity. Here we show that upon viral challenge, rBCG-immunized mice developed a protective humoral immunity, characterized by production of antibodies specific for most hRSV and hMPV proteins. Further, isotype switching from IgG1 to IgG2a was observed in mice immunized with rBCG vaccines and correlated with an increased viral clearance, as compared to unimmunized animals. Finally, sera obtained from animals immunized with rBCG vaccines and infected with their respective viruses exhibited virus neutralizing capacity and protected *naïve* mice from viral replication and pulmonary disease. These results support the notion that the use of rBCG strains could be considered as an effective vaccination approach against other respiratory viruses with similar biology as hRSV and hMPV.

## Introduction

For almost a century, *Mycobacterium bovis* Bacillus Calmette-Guérin (BCG) has been widely used to prevent Tuberculosis and has also been characterized as an effective T helper type 1 (Th1) inducer (1). Further, BCG has been shown to be safe in adults, infants, and newborns. The approach of using BCG as a vector for recombinant expression of heterologous antigens has been previously tested for several pathogens, such as measles virus, rotavirus, hepatitis B virus, *Plasmodium yoelii, Bordetella pertussis*, and *Toxoplasma gondii*, exhibiting promising results in mouse models for those diseases (2–7).

Worldwide, human Respiratory Syncytial Virus (hRSV) is the leading cause of acute lower respiratory tract infections (ALRTIs). HRSV was first identified in 1956 and mainly afflicts infants, young children, elderly, and immunocompromised patients, causing about 34 millions of ALRTIs and ~200,000 deaths per year (8). Next to hRSV, human Metapneumovirus (hMPV) is the second cause of ALRTIs (9) and was first identified in 2001 (10). Although the overall burden in hospitalization remains poorly characterized for hMPV, as it was recently identified, it has been estimated that about a 7–19% of children hospitalization can be due solely to this virus (11, 12). Furthermore, clinical studies have shown that by the age of 5, virtually every child has been infected with both of these viruses, exhibiting classical ALRTI manifestations, which include fever, cough, wheezing, and some clinical manifestation like bronchiolitis, laryngotracheitis, acute bronchitis, and pneumonia (8, 10–14) with an increase in the mucus production, obstruction of bronchoalveolar spaces, exacerbated inflammatory response, and the generation of airway hyper-responsiveness (15–17). In addition, prospective surveillance studies have suggested that children affected by severe hMPV infections usually require longer recovery periods at intensive care units than do children infected with hRSV (11–14). Both hRSV and hMPV are RNA single-stranded, negative sense enveloped viruses, belonging to the *Pneumoviridae* family, particularly the *Orthopneumovirus* genus and the *Metapneumovirus* genus, respectively (15). Furthermore, hRSV has been recently renamed as human Orthopneumovirus (15).

Some reports have suggested that the host immune system is unable to generate an effective and protective immunological memory against either of these viruses, which after disease resolution, prompts the acquisition of repeated infections throughout life (18, 19). Accordingly, it has been described that the nucleoprotein of hRSV (N-hRSV) is able to inhibit the assembly of an effective immunological synapse, apparently by clustering with the pMHC-TCR complex (20). Also, N-hRSV blunts the interferon response by interacting with MDA5 and MAVS, pivotal elements in the main pathways associated with the viral clearance (21). On the other hand, the phosphoprotein of hMPV (P-hMPV) has been described as a crucial component for the assembly of the virus replication core (22). It has been reported that P of hMPV-B1 serotype could interfere with the RIG-I pathway, prompting the inhibition of the interferon I pathway (23). Considering this, both proteins have been previously suggested as possible candidate antigens for the induction of a strong and protective cellular immune response against either hRSV or hMPV infections when used for immunization, respectively (24, 25).

Our group has previously reported that recombinant BCG strains (rBCG) expressing either N-hRSV (rBCG-N) or P-hMPV (rBCG-P) as heterologous antigens, can protect against infection by hRSV or hMPV, respectively (24, 25). In this work, we evaluated the previously unexplored humoral immune response induced in mice immunized with either rBCG-N and rBCG-P. We observed that the post-challenge antibody response is enhanced by the established immunity elicited by both rBCG vaccines (rBCG-N or rBCG-P). This concerted response was able to significantly decrease viral replication and disease by promoting the secretion of neutralizing antibodies specific against the attachment and the fusion glycoproteins of both paramyxoviruses. These results suggest that rBCG strains are good vaccine candidates able to induce a cellular immune response capable of boosting the humoral immune response against unrelated antigens and to prevent the disease cause by both pneumoviruses.

## Materials and Methods

### hRSV and hMPV Propagation and Titration

HEp-2 cells (American Type Culture Collection, CCL-23^TM^) and LLC-MK2 (American Type Culture Collection, CCL-7^TM^) were used to propagate hRSV serogroup A2, strain 13018–8 (clinical isolate obtained from the *Instituto de Salud Pública de Chile*) and hMPV serogroup A, strain CZ0107 (clinical isolate obtained from the *Laboratorio de Infectolog*í*a y Virolog*í*a* of the *Hospital Cl*í*nico, Pontificia Universidad Católica de Chile*) (26, 27). Briefly, cell monolayers were grown in T75 flasks with DMEM (Life Technologies Invitrogen, Carlsbad, CA) supplemented with 10% FBS (Gibco Invitrogen Corp, Carlsbad) for HEp-2 cells and Opti-MEM supplemented with 5% FBS for LLC-MK2 cells, until 80–90% confluence. Flasks containing 5 mL of infection medium [DMEM 1% FBS for hRSV and Opti-MEM 5% FBS medium, supplemented with CaCl_2_ (100 μg/mL) for hMPV] were inoculated with 2 × 10^5^ Plaque formation units (PFU) of the respective virus and incubated at 37°C. After viral adsorption (2 h), supernatants were replaced with fresh medium (DMEM 1% FBS and Opti-MEM) and incubated for 48 h for hRSV and 72 h for hMPV, until visible cytopathic effect was observed. For harvesting, cells were scraped, and the flask content was pooled and centrifuged first at 300 × g for 10 min and then at 500 × g for 10 min in order to remove cell debris. In parallel, supernatants of non-infected cells monolayers (HEp-2 and LLC-MK2) were collected as previously described and used as non-infectious control (Mock). Viral titers of supernatants were determined by immunocytochemistry in 96-well plates with HEp-2 and LLC-MK2 cells monolayers, as previously described (26–29). hRSV and hMPV inoculums were routinely evaluated for lipopolysaccharide and *Mycoplasma* contamination.

### Doses of BCG-WT, rBCG-N, and rBCG-P for Immunization

Vaccine doses of BCG-WT (Danish 1331 strain), rBCG-N, and rBCG-P (both of them obtained as previously described (24, 25) were prepared by growing the mycobacteria on 7H9 liquid medium (Sigma-Aldrich, M0178-500G), supplemented with 10% OADC (Sigma-Aldrich, M0678-1VL) and Kanamycin only for the recombinant bacteria [20 μg/mL] (Sigma-Aldrich, 60615), until reaching an OD600 equal to 0.8. Then, the mycobacteria cultures were washed three times with 1X PBS-0.05% Tween 80, resuspended with 1X PBS-glycerol 50% at a final concentration of 4 × 10^8^ CFU per vial and frozen at −80°C until their use. For immunization, vials were centrifuged at 14,000 g and resuspended in saline solution prior to injection.

### Dot-Blot Assays

Lysates obtained from BCG-WT and rBCG strains expressing either N-hRSV or the P-hMPV, as well as purified N and P proteins as positive controls and 1X PBS as a negative control, were spotted into nitrocellulose membranes. Loaded membranes were incubated for 1 h at 4°C. Membranes were then blocked with a solution 1X PBS and 5% non-fat milk solution for 2 h at RT. Then, membranes were incubated for 1 h with an hRSV anti-N protein monoclonal antibody (1E9/D1 clone 1.48 mg/mL) and hMPV anti-P protein polyclonal antibody (6B12 clone 1.2 mg/mL) diluted in 1X PBS and 5% non-fat milk at a final dilution for both antibodies of 1:750. As a secondary antibody, an HRP-Goat anti-mouse IgG (H+L) (1.5 mg/mL) (Life Technologies, N. Meridian rd., Rockford, IL 61101, USA) was used diluted in 1X PBS, 5% non-fat milk, at a final dilution of 1:2,000, for 1 h. Membranes were washed with 1X PBS, 0.05% Tween 20, three times after every step. Finally, membranes were incubated with the HRP Chemiluminescent Substrate (Invitrogen, Carlsbad, CA 92008, USA) and proteins were visualized with the gel documenter myECL Imager (ThermoFischer Scientific).

### Mouse Immunization and Viral Infection

Six to eight-week-old BALB/cJ mice received a sub-cutaneous injection in the right dorsal flank with 1 × 10^8^ CFU of BCG WT or rBCG strains expressing N-hRSV or P-hMPV, respectively, in a final volume of 100 μL per dose (*n* = 6 per group) (Figures **2A,G**). After 14 days, mice were boosted with the respective BCG strain. Twenty-one days post immunization, mice were intraperitoneally anesthetized with a mixture of ketamine and xylazine (80 and 4 mg/kg, respectively), and challenged by intranasal instillation with ~1 × 10^7^ PFU of hRSV A2, strain 13018-8 or ~1 × 10^6^ PFU of hMPV A, strain CZ0107, accordingly to the vaccine injected, in a final volume of < 100 μL per mouse. Blood samples were obtained from these animals before immunization, boost, challenge, and at 7 days post-infection (dpi) and 14 dpi. For hRSV infected mice, lung samples, and Bronchoalveolar Lavage (BAL) were obtained at 7 and 14 dpi. For hMPV-infected mice, blood samples, lung samples, and BAL were obtained at 28 dpi, and 21 days post re-infection with ~1 × 10^6^ PFU of hMPV (49 dpi). All animals were treated and manipulated with supervision of a veterinarian and according to the institutional guidelines of the Pontificia Universidad Católica de Chile and the “Guide for the care and use of laboratory animals”.

### Quantification of IgG Isotypes

Ninety-six well ELISA plates were separately coated with the following antigens overnight at 4°C: 50 μL/well of hRSV or hMPV (previously UV-inactivated for 45 min and sonicated by 10 min to expose as many antigens as possible), 100 ng/well of N-hRSV purified protein or 100 ng/well of F-, G- and P-hRSV proteins (SinoBiological, Beijing, China). For hMPV ELISA, plates were coated with 200 ng/well of P-, M-, and M2.1- proteins purified.

Plates were blocked with 200 μL of 1X PBS, 2% Fish gelatine. After 1 h at RT, plates were washed three times with 200 μL of 1X PBS, 0.05% Tween 20 and incubated for 1 h at RT with 100 μL of the different serum samples previously diluted at 1:500 in triplicate (14 dpi for hRSV and 28 dpi for hMPV). Then, the plates were washed three times and incubated with 50 μL of 1:2,000 dilution of HRP-Goat anti-mouse IgG (H+L) (Life Technologies, N. Meridian rd., Rockford, IL 61101, USA) for 1 h at RT. Afterwards, plates were washed and revealed with 50 μL of 1 mg/mL 3-39-5-59-tetramethylbenzidine (TMB, Merck) at RT in the darkness. After 10 min, 50 μL of H_2_SO_4_ solution were added to stop the reaction. Plates were analyzed in an ELISA reader at 450 nm (Multiskan Ex, Thermo Labsystems).

Immunoglobulin isotypes were also analyzed from the same sera samples in similar conditions. 96-well ELISA plates were coated overnight at 4°C with 50 μL of hRSV or hMPV, previously UV-inactivated by 45 min and sonicated by 10 min. After blocking and washing as previously described, the plates were incubated with 50 μL of 1:500 dilution of sera sample in triplicate (14 dpi for hRSV and 49 dpi for hMPV) 1 h at RT. Biotinylated Rat anti-Mouse IgG2a (Clone RMG2a-62, Biolegend, San Diego, CA) and IgG1 (Clone RMG1-1, Biolegend, San Diego, CA) antibodies were used to assess the titers of circulating anti-hRSV IgG isotype in vaccinated and control mice. Plates were read at 450 nm and data was represented as a ratio of the IgG2a concentration/IgG1 concentration. The concentration of different IgG, IgG1, and IgG2a isotypes, was measured interpolating the absorbance values in a curve of mice purified IgG-antibody. The production of the hybridomes was made by GrupoBios, Chile.

### Determination of Linked Recognition Mechanism

Six to eight-week-old BALB/cJ mice received a sub-cutaneous injection in the right dorsal flank with 1 × 10^8^ CFU of either BCG WT or rBCG-N strains (*n* = 5 for each group). Other mice were immunized with purified N-hRSV protein in Freund adjuvant or purified N-hRSV protein in Aluminum Hydroxide adjuvant (Supplementary Figure 2). After 14 days, animals were boosted with the same dose and at day 21 post-immunization, mice were euthanized. Spleens were collected and plated at a final concentration equal to 1 × 10^6^ cells/mL in 24 well plates. Then, cells were stimulated with purified N-hRSV protein (1 μg/mL) and after 72 h T cells were purified by MACS columns following the manufacturer instructions (mouse CD4^+^ T cell isolation kit, Miltenyl Biotec). Purified T cells were transferred to a total of 5 *naïve* mice per group and 1 day post-transfer, animals were either treated with mock or infected with hRSV. Animal body weight loss was monitored and at 7 dpi mice were euthanized (Data not shown). Sera samples were used to measure antibody secretion against the whole virus and the N, F, and G hRSV proteins by indirect ELISA, as described above.

### Serum Neutralization Assays

HEp-2 or LLC-MK2 cells were grown until 80–90% of confluence. Then, the sera from 14 dpi in the hRSV-infected animals or 21 days post-second infection (49 dpi) in the hMPV-infected animals were pooled and incubated at 56°C for 30 min to inactivate the complement system. Sera were then incubated with GFP-recombinant viruses (hRSV or hMPV), respectively, at 37°C for 1 h. The mixtures were added to the respective cell culture and incubated for 1 h at 37°C in 5% of CO_2_ supplement. Then supernatants were replaced with fresh DMEM 1% FBS for the HEP-2 cells and Opti-MEM for the LLC-MK2 cells. Cells were incubated for 48 h at 37°C with a 5% of CO_2_ supplement. GFP expression in both cells was visualized by epifluorescence microscopy and the plaque-forming units (PFU) were quantified as previously described (30). As a positive control, cells were treated with GFP-expressing virus without sera, and as negative control cells were treated with Mock.

### Passive Transfer of Immune IgG From rBCG-Vaccinated Mice to Naive Animals

Six to eight-week-old BALB/cJ mice were intravenously transferred with 100 μg of total IgG from the sera of the following animal groups: non-immunized but hRSV-infected (nt-hRSV), BCG-WT immunized hRSV-infected, rBCG-N immunized hRSV-infected, non-immunized but hMPV-infected (nt-hMPV), BCG-WT hMPV-infected, and rBCG-P immunized hMPV-infected mice (*n* = 6 for each group). Serum samples used for these experiments were collected at 14 dpi (hRSV) and 49 dpi (hMPV). One day after the transfer, animals were intraperitoneally anesthetized with a mixture of ketamine and xylazine (80 and 4 mg/kg, respectively) and challenged by intranasal instillation with 1 × 10^7^ PFU of hRSV or 1 × 10^6^ PFU of hMPV, respectively. In both cases, Mock treatments were used as negative control and virus infected non-transferred (nt) as an infection-control. In order to evaluate the effect of the protection, animals were immunized with 100 μg of palivizumab (intraperitoneal route) as a positive antiviral control against hRSV. Weight loss was daily measured in both experiments (Data not shown). At 7 dpi, animals were terminally anesthetized by i.p. injection of a mixture of ketamine and xylazine (20 and 1 mg/kg, respectively) and BALs and lungs were collected.

### Evaluation of hRSV- and hMPV-Associated Disease Parameters

To determine the infiltration of polymorphonuclear cells, BALs were collected as previously described (24). Briefly, lung samples were collected, clamping the non-lobed section of the lung, stored in 4% PFA and later used to histological analyzes, as previously described (24). A lower section of the lobate lung was collected and stored in TRIzol (Life Technologies), and the RNA was extracted following the manufacture conditions. The rest of the lung was collected and incubated with collagenase IV for 30 min at 37°C, then PBS-1X/1%FBS was added to stop the collagenase reaction. Lung samples were homogenized using a 70 μm cell strainer (BD Biosciences) and centrifugated at 0.3 g for 5 min at 4°C. Supernatants were discarded, and pellets were incubated for 5 min with an ACK solution at RT. The cells were washed with PBS/1%FBS and centrifuged once again. Finally, samples were resuspended in 1 mL of PBS/1%FBS and used for flow cytometry. BAL samples were collected by gently instilling 1 mL of PBS with a tuberculin syringe through the trachea of the animals and subsequently recovered (twice), while the lung was pinched. The final volume was centrifuged at 0.3 g for 5 min at 4°C. Supernatant was stored at −80°C and pellets were resuspended in 300 μL of PBS/1%FBS. Finally, the cells from Lungs and BALs were incubated in 96 six-well plates and centrifugated at 0.3 g for 5 min at 4°C, then the samples were stained with anti–CD11b PerCP-Cy 5.5 (clone M1/70, BD Pharmingen), anti–CD11c APC (clone HL3, BD Pharmingen), and anti-Ly6G FITC (Clone 1A8, BD Pharmingen) antibodies. Data were acquired on FACSCanto II cytometer (BD Biosciences) and analyzed using FlowJo v10.0.7 software (BD Biosciences). Viral loads were detected in the lungs by qRT-PCR as previously described (24, 25). Also, lung samples were stored in 4% PFA, maintained at 4°C, embedded in paraffin, cut, and stained with H&E as previously described (24).

### Statistical Analyses

All statistical analyses were performed using GraphPad Prism version 6.0 Software. Statistical significance was assessed using One-way ANOVA with a posteriori Tukey test or Two-way ANOVA test with a posteriori Tukey test.

## Results

### rBCG-N and rBCG-P Vaccines Are Able to Reduce Disease-Associated Parameters Caused by hRSV or hMPV Infection

The expression of the hRSV N protein or the hMPV P protein in rBCG-N or rBCG-P strains, respectively, was corroborated by dot blot assays. Both recombinant BCG strains expressed significant amounts of the respective proteins, as compared to negative and BCG-WT controls (Supplementary Figure 1).

**Figure 1:**
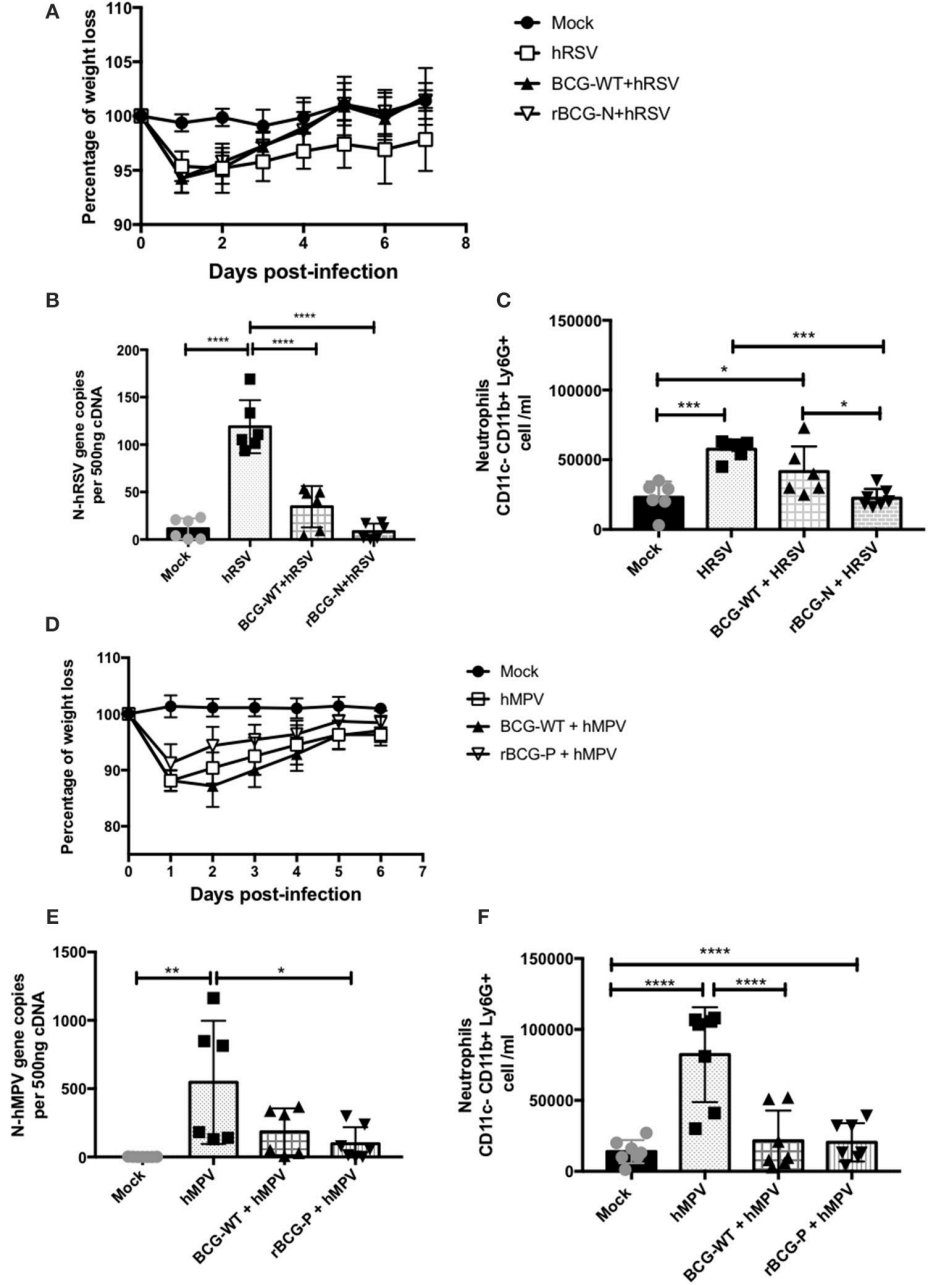
Evaluation of infection parameters from animals infected with hRSV or hMPV. Several infection parameters were measured in the mice infected with hRSV or hMPV to corroborate that both viral infections promote the diseases. For hRSV all the parameters were measured at day 7 post-infection, for hMPV weight loss was measured for 7 days post-infection, while the neutrophils infiltrate and the viral load were measured at day 28 pi. The weight loss was measured from hRSV- **(A)** and hMPV-infected mice **(D)**. Also, the viral load was quantified for hRSV **(B)** and **(E)** hMPV. In addition, the neutrophils infiltration was measured in the hRSV- **(C)** and hMPV-infection **(F)**, respectively. The bars observed in the **(B,C)** are accordingly: 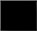 Mock-treated, 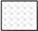 hRSV-infected, 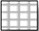 Immunized with BCG-WT and infected with hRSV (BCG-WT+hRSV) and 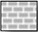 Immunized with rBCG-N and infected with hRSV (rBCG-N +hRSV). The bars observed in **(E,F)** are accordingly: 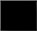 Mock-treated, 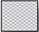 hMPV-infected, 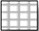 BCG-WT +hMPV, and 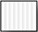 rBCG-P + hMPV. *N* = 3 for each group, with two independent experiments. Differences were evaluated by a one-way ANOVA (*p < 0.05; **p < 0.01; ***p ≤ 0.001; ****p ≤ 0.0001). The error bars represent the Standard Deviation (SD).

**Figure 2:**
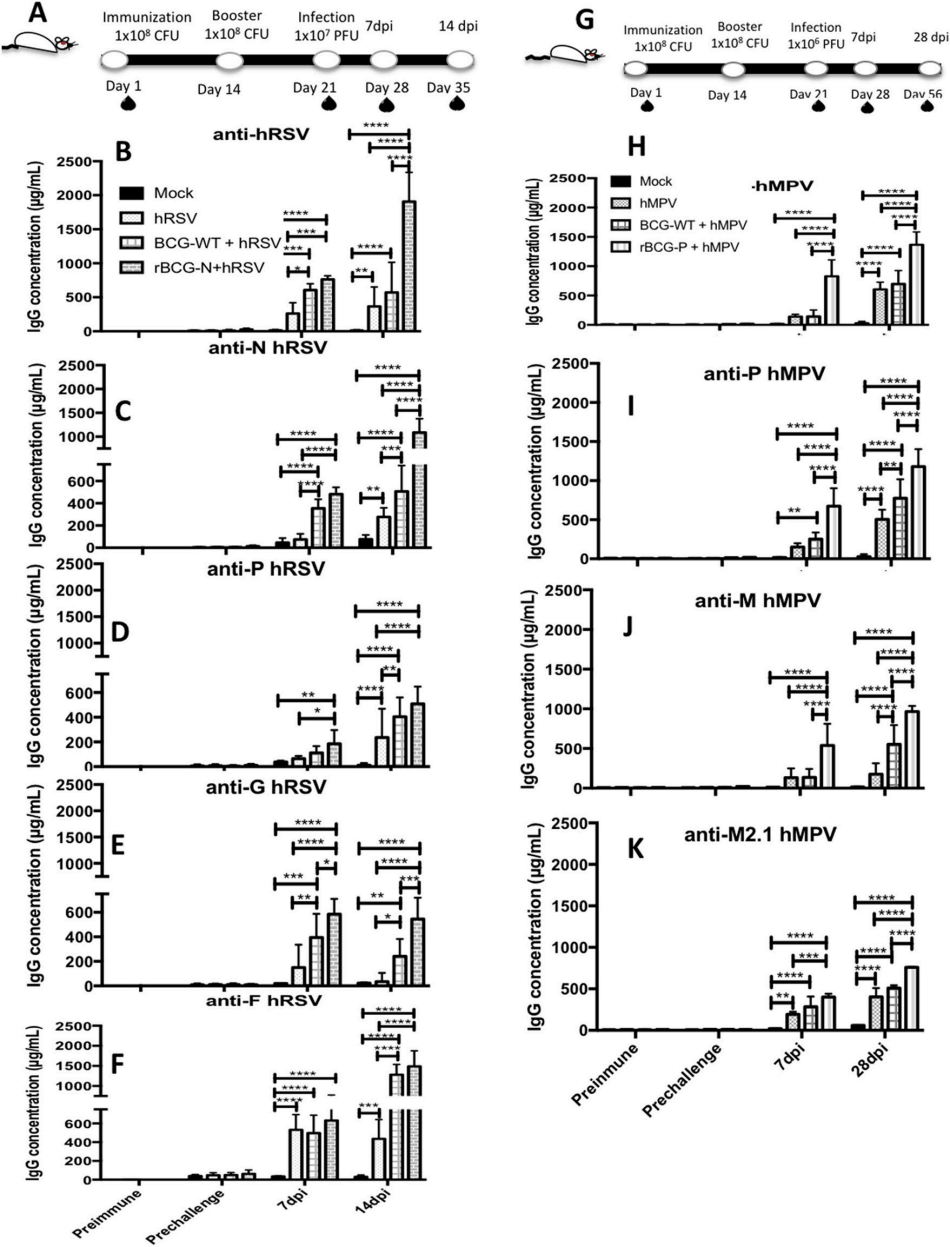
Evaluation of the induction of IgG specific-antibodies against viral antigens after a viral infection. IgG for different antigens were evaluated from the sera obtained at different time points: Prior to immunization (Preimmune), prior to virus infection (Prechallenge), 7 and 14 days post-infection (7 dpi and 14 dpi) for hRSV or 7 dpi and 28 dpi for hMPV, respectively **(A, G)**. Specific antibodies levels were determined by ELISA assay against hRSV **(B)**, N protein **(C)**, P protein **(D)**, G protein **(E)**, F protein **(F)**. Similarly, this same ELISA assays were done against hMPV **(H)**, P protein **(I)**, M2.1 protein **(J)**, and M protein **(K)**. The bars observed in the **(B–F)** are accordingly: 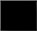 Mock-treated, 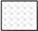 hRSV-infected, 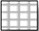 BCG-WT +hRSV, and 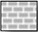 rBCG-N +hRSV. On the other hand, the bars observed in **(H–K)** are accordingly: 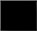 Mock-treated, 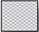 hMPV-infected, 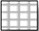 BCG-WT +hMPV, and 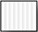 rBCG-P +hMPV. The measure was made at 450 nm. The sera from 6 different animals for each group was used for the ELISA assay, with each serum tested in 3 replicates. *N* = 3 for each group, with two independent experiments. Differences were evaluated by a two-way ANOVA (*p < 0.05; **p < 0.01; ***p ≤ 0.001; ****p ≤ 0.0001). The error bars represent the Standard Deviation (SD).

The hRSV- and hMPV-associated disease parameters were evaluated post-immunization to evaluate the protective capacity of the rBCG strains against each respective viral infection (Figure 1). The immunization scheme is described at the Materials and Methods section (Mouse immunization and viral infection) and is also shown in Figure **2A**. As shown in Figure 1A, infection with 1 × 10^7^ PFU of hRSV induces body weight loss in all infected animals 1 day post-infection (dpi). Importantly, rBCG-N-immunized and hRSV-infected mice started recovering their original body weight at 2 dpi and recovered their original body weight by day 5 pi. On the contrary, the non-immunized and hRSV-infected group (hRSV) was not able to recover their initial weight by 7 dpi.

Likewise, as seen for the rBCG-N immunized and hRSV-infected mice (rBCG-N+hRSV), vaccination was able to drastically decrease viral loads in the lungs of the hRSV-infected animals as compared to the non-immunized but infected group (hRSV) (Figure 1B). Further, the BCG-WT immunized and hRSV-infected mice (BCG-WT+ hRSV) showed a significant decrease in the viral loads, as compared to hRSV mice, although this decrease was less pronounced than the observed for the rBCG-N+hRSV mice (Figure 1B). Remarkably, the number of BAL neutrophils (CD11b^+^ Ly6G^+^ cells) infiltrating the lungs of rBCG-N-vaccinated animals was lower than those vaccinated with BCG-WT or hRSV-infected, naïve mice (Figure 1C).

A similar protective response was found for the hMPV-infected animals with a similar body weight loss and recovery (Figure 1D). Also, the rBCG-P immunized and hMPV-infected mice (rBCG-P+hMPV) showed a significant decrease in viral loads as compared to the non-immunized and hMPV-infected mice (hMPV) (Figure 1E) and a decrease in the number of infiltrating neutrophils in the BALs (Figure 1F). Remarkably, no significant differences were detected in the BALs of BCG-WT+hMPV when compared with the hMPV-infected naive mice. Therefore, and as described previously, immunization with recombinant rBCG vaccines significantly decrease disease parameters associated to hRSV and hMPV infection, such as body weight loss, viral loads, and neutrophil infiltration in the lungs.

### Immunization With rBCG Strains Enhances Post-challenge Antibody Responses for Several Viral Proteins

To evaluate whether the immune protection induced by the rBCG vaccines involves a humoral-mediated response, the presence of anti-hRSV or -hMPV antibodies in sera from immunized mice (BCG-WT, rBCG-N, and rBCG-P groups) was measured (Figure 2). An increase in the total anti-hRSV antibodies was observed in the rBCG-N+hRSV sera at both 7 and 14 dpi, as compared to all the other treatments (Figure 2B). BCG-WT+hRSV treated mice showed a significant increase in anti-hRSV antibodies as compared to mock-treated and the hRSV-infected mice at 14 dpi (Figure 2B). However, this increase was not as pronounced as the one observed for the rBCG-N+hRSV treated mice. No significant differences were detected between hRSV- and the mock-treated animals at day 7 post-infection, although there is a clear tendency to an increase in the quantity of IgG.

Since the production of total antibodies against hRSV was significantly higher in infected mice previously immunized with rBCG-N, we measured the specific antibody production against various individual hRSV proteins, such as N, P, G, and F proteins (Figures 2C–F). We observed that at 7 and 14 dpi rBCG-N+hRSV treated mice secreted more anti-N antibody as compared with all the other groups (with the exception of the BCG-WT+hRSV group at 7 dpi). At 14 dpi BCG-WT+hRSV also showed an increase in the anti-N antibody secretion, as compared to mock-treated and the hRSV mice (Figure 2C). A significantly higher antibody secretion against P-, G-, and F-hRSV proteins was detected at 7 and 14 dpi for the rBCG-N+hRSV treated mice as compared to the mock controls (Figures 2D–F). Remarkably, this increase was statistically higher when compared to all the other treatments at 14 dpi, with the exception of BCG-WT+hRSV for the F-protein (Figure 2F). The IgG concentration for the F protein was as high as the one detected for the total hRSV antibodies (about 1,500–2,000 μg/mL), indicating that this protein is one of the major antigenic determinants of the virus, accordingly to what has been previously described (31).

When the humoral response induced by the rBCG-P vaccine was evaluated, high antibody titers were observed starting from 28 dpi against total hMPV, P-, M2.1-, and M-proteins for the rBCG-P+hMPV, when compared with all the other treatments, with the exception of the BCG-WT+hMPV treated mice for the total anti-hMPV (Figures 2H–K). Likewise, the BCG-WT+hMPV treated mice also showed significantly higher anti-hMPV antibody titers as compared to the mock-treated mice at 28 dpi (Figure 2H). Remarkably, a higher secretion of anti-M antibodies was detected for the rBCG-P+hMPV treated mice as early as 7 dpi, as compared to all the other treatments (Figure 2J). Finally, the BCG-WT+hMPV treated mice and the hMPV infected naïve mice showed higher levels of anti-M2.1 antibodies as compared to the mock treated-mice at 28 dpi (Figures 2I–K). Again, post-infection IgG levels for all the proteins evaluated was significantly higher in rBCG-P-immunized mice as compared to the other groups, suggesting that the effect was induced by both vaccination and infection.

The low IgG levels detected in the sera from mock-treated mice against both G and F proteins, could be associated with the assay methodology properties, as ELISA may have shown unspecific interactions (Figure 2). These results suggest that immunization with rBCG vaccines, upon challenge with the pathogen, is able to promote the secretion of antibodies against several proteins of each respective virus.

### Transfer of Activated N-hRSV Protein-Specific T Cells From Immunized Mice to Challenged Mice Leads to Diversification of the Antibody Response Against Viral Proteins

**Figure 3:**
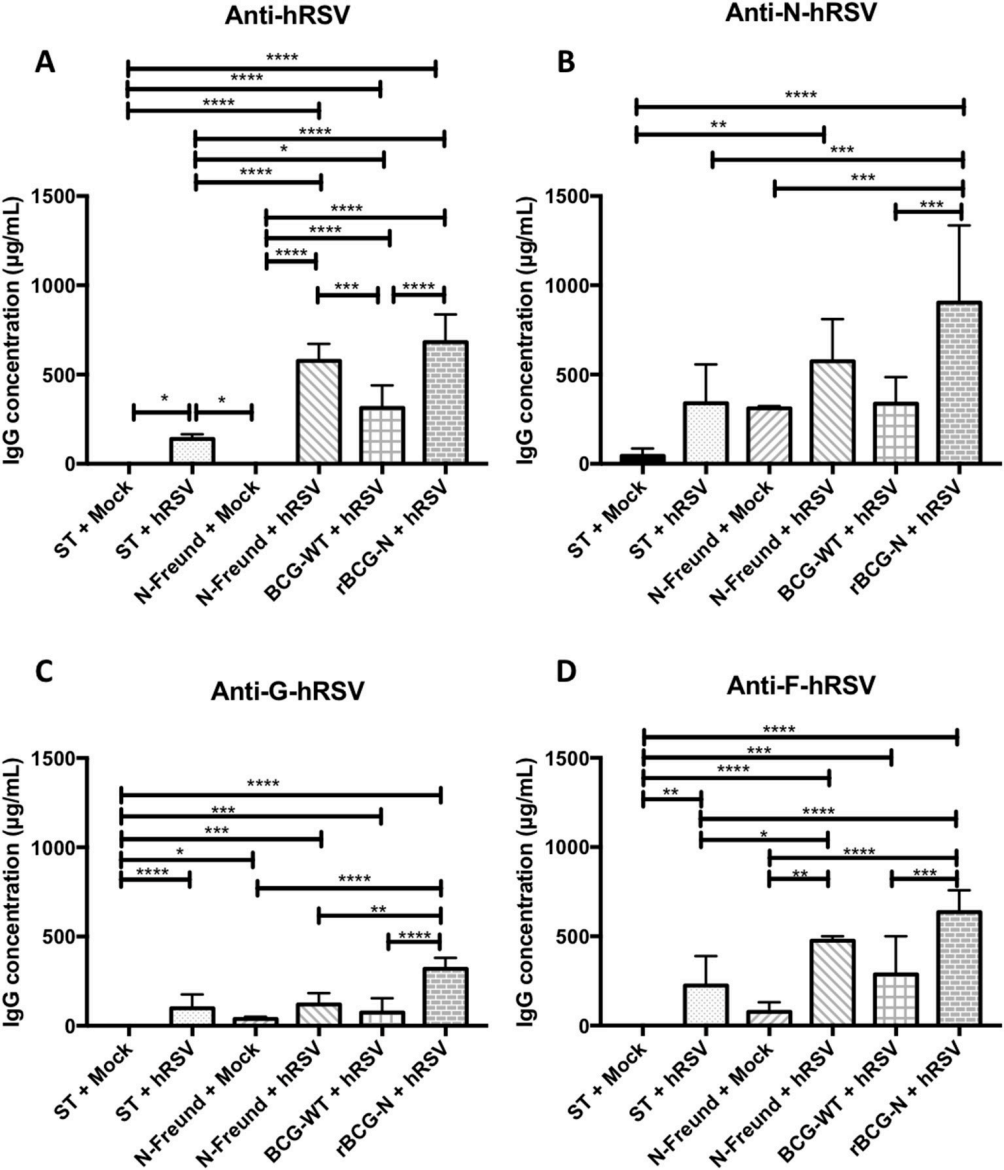
The rBCG-N vaccine promotes the secretion of antibodies against several antigens through the Linked Recognition mechanism. The secretion of specific antibodies against the whole virus and the N, F, and G hRSV proteins were measured by indirect ELISA from the sera collected of the T cell transferred and infected mice after 7 dpi. The anti- hRSV **(A)**, anti-N-hRSV **(B)**, anti-F-hRSV **(C)** and anti-G-hRSV **(D)** were evaluated and measured at 450 nm. The bars observed in the **(A–D)** are accordingly: 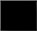 Non-transferred and Mock-treated (ST+Mock), 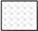 Non-transferred, and hRSV-infected (ST+hRSV), 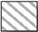 T cells transferred from mice immunized with hRSV-N protein in Freund adjuvant, treated with mock (N-Freund+Mock), 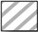 T cells transferred from mice immunized with hRSV-N protein in Freund adjuvant, infected with hRSV(N-Freund+hRSV), 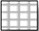 T cells transferred from mice immunized with BCG-WT and then infected with hRSV (BCG-WT + hRSV) and 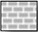 T cells transferred from mice immunized with rBCG-N and then infected with hRSV (rBCG-N +hRSV). The sera from five different animals for each group was used for the ELISA assay, with each serum tested in three replicates. Data combinate from two independent experiment for ST+mock, ST+hRSV, BGC-WT+hRSV and rBCG-N+hRSV groups (*N* = 9), and one independent experiment for N-Freund+Mock, N-Freund+hRSV groups (*N* = 5). Differences were evaluated by a one-way ANOVA (*p < 0.05; **p < 0.01; ***p ≤ 0.001; ****p ≤ 0.0001). The error bars represent the Standard Deviation (SD).

As shown above, a significant increase in the secretion of antibodies specific against proteins different than those expressed by the rBCG vaccines was detected upon immunization and viral challenge (Figure 2).Therefore, to elucidate whether the secretion of antibodies against the different proteins was an effect of either the expressed proteins by themselves or an effect of the vaccine prototypes as a whole, we measured the secretion of antibodies by hRSV-infected mice that were previously adoptively transferred with T cells derived from rBCG-N immunized mice (Figure 3 and Supplementary Figure 3). Briefly, mice were immunized with 1 × 10^8^ CFU of either BCG-WT or rBCG-N, then at day 14 post-immunization mice were boosted with the same vaccine dose. Unimmunized or mice immunized with purified hRSV Nucleoprotein with either Freund adjuvant or Aluminum Hydroxide adjuvant, were included as controls (Supplementary Figure 2). At day 21 after the first immunization, animals were euthanized, spleens were collected and 72 h-long splenocyte cultures were performed stimulating with purified hRSV nucleoprotein. Next, T cells were purified and immediately transferred to *naïve* mice. Animals were then infected and euthanized at 7 dpi, sera were collected, and the secretion of specific antibodies was measured.

We detected that mice receiving T cells from the rBCG-N immunized group and then infected with hRSV (rBCG-N+hRSV) induced the higher antibody secretion against the proteins evaluated, as compared to control animals (Figure 3). The anti-hRSV antibody titers were higher in the rBCG-N-transferred mice, exhibiting a significant increase when compared to most of the other groups, except the one immunized with purified hRSV-N protein with Freund as adjuvant and then infected with hRSV (N-Freund+hRSV), were no significant differences were detected. Remarkably, mice receiving T cells from the BCG-WT immunized and then infected with hRSV (BCG-WT+hRSV) showed no significant differences as compared to the N-Freund group (Figure 3A).

A similar response was observed when the anti-N antibody secretion was evaluated, as only rBCG-N+hRSV transferred mice showed a rise in antibody titers, as compared to all other experimental groups. No significant differences were observed between the mock-treated, hRSV-infected and BCG-WT+hRSV transferred groups (Figure 3B). Regarding the other viral proteins, we identified an increase in the anti-F and anti-G antibody secretion for the rBCG-N+hRSV transferred mice as compared to mock-treated mice, whether they were immunized or not (Figures 3C,D).

As controls, we also included animals immunized with the nucleoprotein of hRSV, along with either the Freund Adjuvant—a Th-1 inducer—or Aluminum hydroxide Adjuvant—a Th-2 inducer (32, 33) (Supplementary Figures 2, 3). Remarkably, the total anti-hRSV, anti-F and anti-G antibodies secretion was statistically higher in the mice transferred with the T cells from the animals immunized with the Aluminum hydroxide adjuvant, as compared to all the other groups. This effect was not seen when the specific anti-N antibodies were measured, as the rBCG-N+hRSV transferred animals exhibited a significantly higher antibody secretion as compared to all the groups except for the Aluminum hydroxide (Supplementary Figure 3). These results suggest that, although animals are being immunized with a particular antigen, the vaccine elicited T helper response seems to promote the activation of B cells specific for viral antigens beyond those encoded by these recombinant proteins. As such, these data also suggest the normal presentation of nucleoprotein and phosphoprotein peptides by the B cells of hRSV and hMPV infected mice, respectively.

### rBCG Vaccines Induce an Efficient Antibody Class Switching Upon Viral Infection

**Figure 4:**
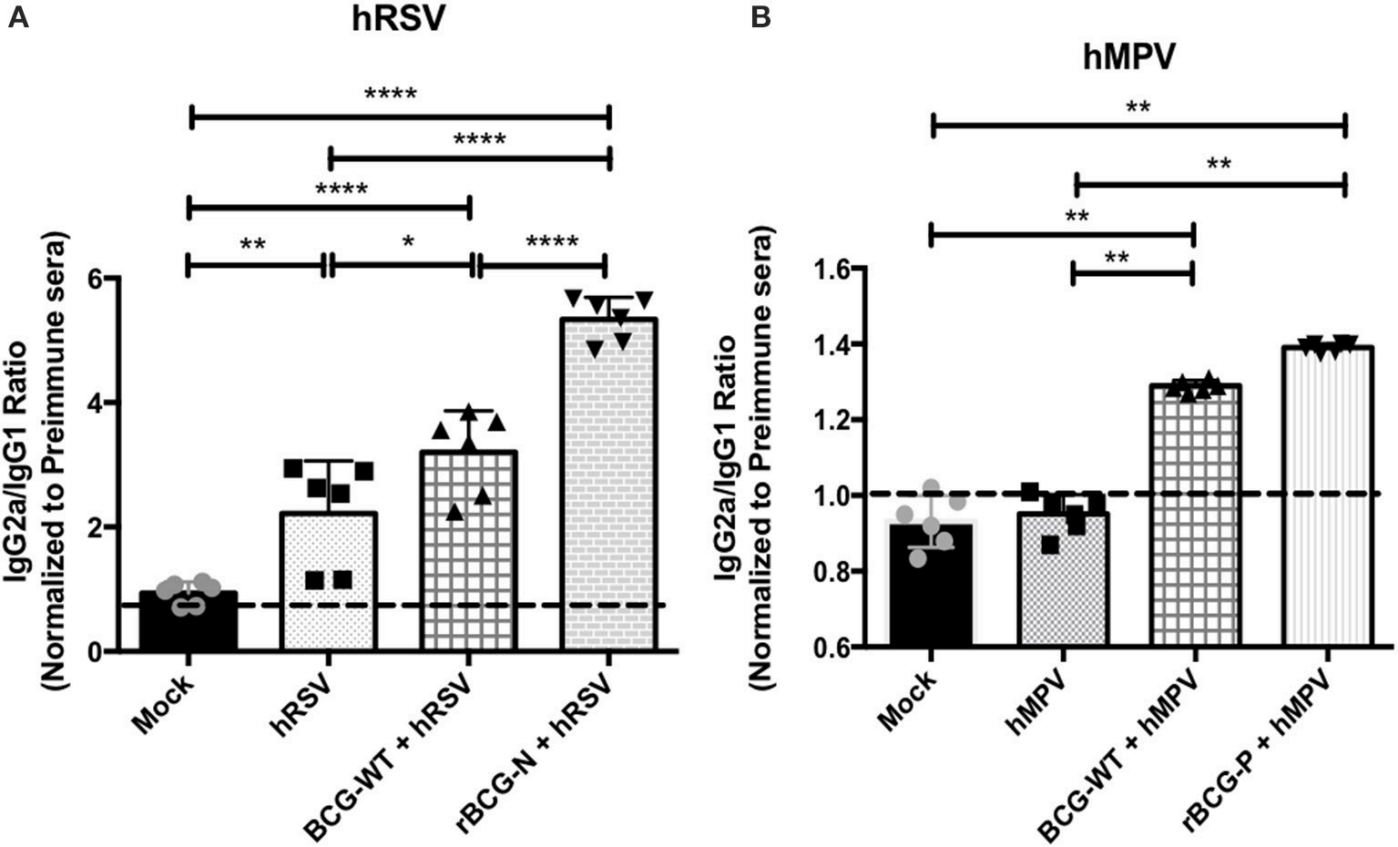
The rBCG strains induce a strong IgG2a isotype switching after a viral infection. The secretion of specific antibody isotype IgG2a and IgG1 was measured in both vaccines by indirect ELISA. The assay was done using a total virus extract and expressed as the ratio between the IgG2a and IgG1 in animals infected with hRSV **(A)** and hMPV **(B)**. Secretion of this specific antibody was measured from sera at 14 dpi (hRSV) or 49 dpi (hMPV) using total extract of each virus to incubate the plate. The bars observed in the **(A)** are accordingly: 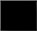 Mock-treated, 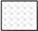 hRSV-infected, 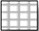 BCG-WT +hRSV, and 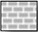 rBCG-N +hRSV. On the other hand, the bars observed in **(B)** are accordingly: 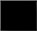 Mock-treated, 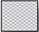 hMPV-infected, 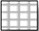 BCG-WT +hMPV, and 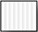 rBCG-P +hMPV. The measure was made at 450 nm. The sera from six different animals for each group was used for the ELISA assay, with each serum tested in three replicates. *N* = 3 for each group, with two independent experiment. Differences among groups were evaluated by a one-way ANOVA (*p ≤ 0.05; **p ≤ 0.01; ***p ≤ 0.001; ****p ≤ 0.0001). The error bars represent the Standard Deviation (SD).

Since antibody class switching also depends on the help provided by CD4^+^ T cells, we sought to evaluate whether this phenomenon is also promoted by the cellular immunity induced by rBCG vaccination. Sera obtained from the experimental groups presented in Figure 2 were analyzed by ELISA and the IgG2a/IgG1 antibody ratio was determined for virus-specific antibodies. Similar responses were observed for both rBCG vaccines as compared with their respective control groups (Figure 4). rBCG-N+hRSV treated mice showed the highest IgG2a/IgG1 ratio for virus-specific IgGs as compared to all of the others control mice (Figure 4A). Nevertheless, the BCG-WT+hRSV treated mice also showed an increased IgG2a/IgG1 ratio as compared to mock-treated and hRSV mice, although this rise was not as high as the observed for the sera derived from rBCG-N+hRSV-treated mice (Figure 4A). Additionally, the hRSV infected naïve mice only showed an increase in the antibody ratio when compared with the mock-treated animals (Figure 4A).

As stated above, a similar pattern was observed in the sera derived from rBCG-P-vaccinated mice (Figure 4B). The rBCG-P+hMPV treated mice also showed an increase in the IgG2a/IgG1 antibody ratio, as compared with mock-treated and hMPV infected naïve mice (Figure 4B). Further, a higher IgG2a/IgG1 antibody ratio was observed for the BCG-WT+hMPV treated mice as compared to hMPV-infected and mock-treated mice. Remarkably, no differences were detected in the ratio among the sera from mock-treated and hMPV infected naïve mice. These results indicate that antibodies secreted by animals immunized with rBCG vaccines undergo significant isotype switching that may contribute to their viral clearance capacity.

### rBCG-Induced Antibodies Show Virus Neutralizing Capacity *ex vivo*

**Figure 5:**
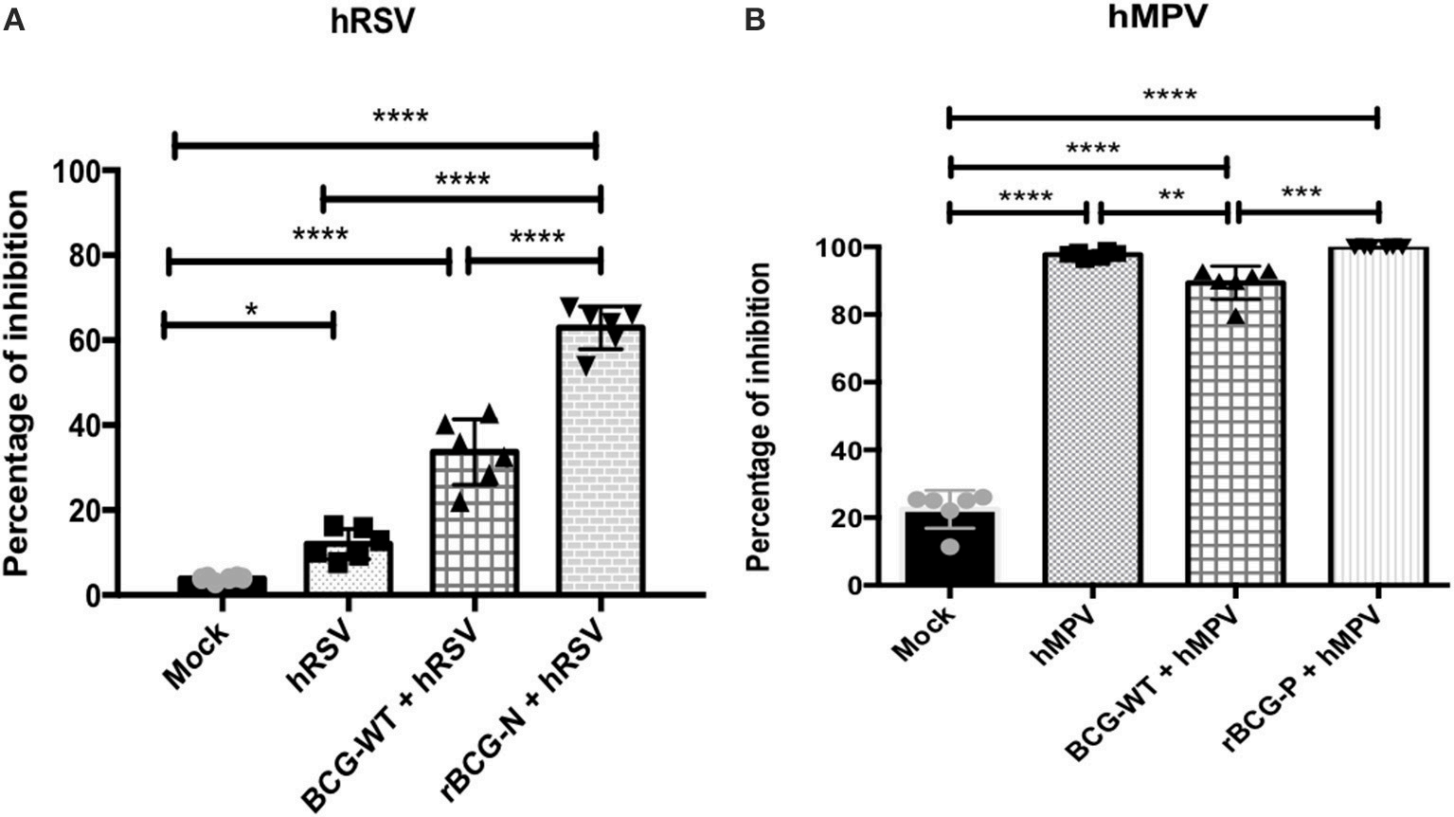
Sera from immunized mice are able to neutralize both viruses *in vitro*. Sera pools from all experimental groups were incubated with the GFP-virus and then used to infected cells to evaluate whether they have the ability to neutralize viral replication. The effect was measured counting the plaques forming units (PFUs) induced by each GFP- virus and indicated as percentage of inhibition. The effect observed from the sera of the hRSV-infected animals was evaluated at 14 dpi **(A)** and the effect from hMPV-infected animals was evaluated at 49 dpi **(B)**. The bars observed in the **(A)** are accordingly: 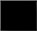 Mock-treated, 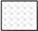 hRSV-infected, 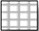 BCG-WT +hRSV, and 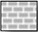 rBCG-N +hRSV. On the other hand, the bars observed in **(B)** are accordingly: 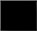 Mock-treated, 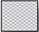 hMPV-infected, 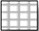 BCG-WT +hMPV, and 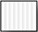 rBCG-P +hMPV. *N* = 3 for each group, with two independent experiments. Differences were evaluated by a one-way ANOVA (*p ≤ 0.05; **p ≤ 0.01; ***p ≤ 0.001; ***p ≤ 0.0001). The error bars represent the Standard Deviation (SD).

To evaluate whether the antibodies generated by the rBCG immunization are able to neutralize hRSV or hMPV replication *ex-vivo*, sera collected from the immunized and infected animals were incubated with hRSV or hMPV and later used to infect cells (Hep-2 for hRSV and LLC-MK2 for hMPV), then PFUs were evaluated (Figure 5). Sera obtained from rBCG-N+hRSV-treated, BCG-WT+hRSV-treated and hRSV-infected mice led to a decrease in the number of viral PFUs, represented as an increase in the percentage of inhibition of viral loads, as compared to mock-treated mice. Furthermore, sera from rBCG-N+hRSV-treated mice showed a higher antiviral effect when compared with sera derived from hRSV-infected and BCG-WT+hRSV-treated animals (Figure 5A). As for hMPV-infected animals, rBCG-P+hMPV-treated, BCG-WT+hMPV-treated, and hMPV-infected mice showed a significant increase in the percentage of inhibition, as compared to mock-treated mice. Moreover, rBCG-P+hMPV-treated and hMPV-infected mice showed significantly higher percentage of inhibition as compared to sera from BCG-WT+hMPV-treated animals. However, no differences were found among rBCG-P+hMPV and hMPV mice (Figure 5B). These data suggest that immunization with rBCG promotes the secretion of antibodies with enhanced capacities to neutralize virus in cell culture experiments.

Antibodies triggered by rBCG vaccination followed by virus challenge protect *naïve* mice from viral-induced lung pathology.

**Figure 6:**
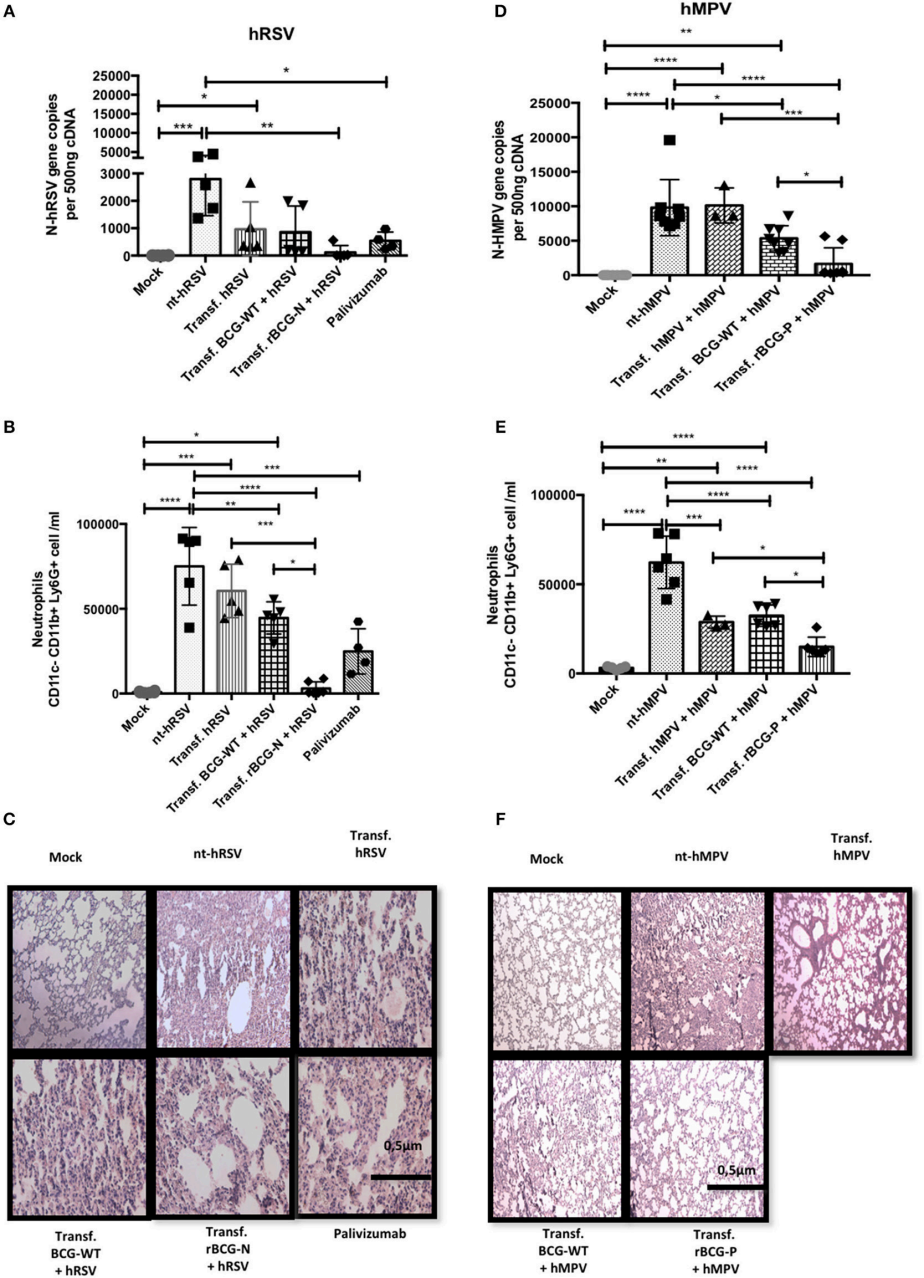
Passive immunity transfer from sera of previously rBCG immunized mice protects naïve mice from hRSV- and hMPV-associated pathology. Sera from 14 dpi (hRSV) or 49 dpi (hMPV) was passively transferred to naïve mice. Then mice were then infected with hRSV or hMPV, respectively. As a control, a non-transferred group was also infected (nt-hRSV or nt-hMPV). Parameters as viral load in lung by RT-qPCR **(A** for hRSV and **D** for hMPV), neutrophils infiltrated in BALs **(B** for hRSV and **E** for hMPV) and histopathological lung H&E staining **(C** for hRSV and **F** for hMPV) were evaluated. For hRSV, Palivizumab was used as a positive control. The bars observed in the **(B,C)** are accordingly: 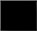 Mock-treated, 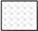 nt-hRSV, 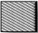 Transf. hRSV, 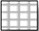 Transf. BCG-WT +hRSV, and 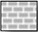 Transf. rBCG-N +hRSV and 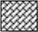 Palivizumab. On the other hand, the bars observed in **(E,F)** are accordingly: 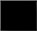 Mock-treated, 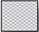 nt-hMPV, 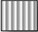 Transf. hMPV 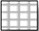 Transf. BCG-WT +hMPV, and 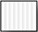 Transf. rBCG-P +hMPV. Differences were evaluated by a one-way ANOVA (*p ≤ 0.05; **p ≤ 0.01; ***p ≤ 0.001; ****p ≤ 0.0001). These results are from two independent experiments with 3 animals by group. The error bars represent the Standard Deviation (SD).

After evaluating the neutralizing effect of the sera obtained from the immunized mice *ex-vivo*, we sought to evaluate the capacity of these sera to protect from a viral infection *in vivo*. With this aim, sera were transferred from immunized to *naïve* mice, which in turn were challenged with the respective virus. As shown in Figure 6A, a significant viral load reduction was observed for mice transferred with sera from rBCG-N+hRSV-treated mice as compared to non-transferred but hRSV-infected mice (nt-hRSV). The adoptive transfer of sera from rBCG-N+hRSV-treated mice led to similar levels of protection as compared to the transfer of Palivizumab, a commercially available humanized anti-hRSV-F mAb (Figure 6A). Further, a significant decrease in the infiltration of neutrophils to the lungs was observed for mice that had received sera from rBCG-N+hRSV-treated animals (Figure 6B).

Additionally, H&E staining of histopathological lung samples showed that nt-hRSV and hRSV animals exhibited a loss of lung structure and a high cellular infiltration (Figure 6C). In contrast, animals transferred either with Palivizumab, sera from BCG-WT+hRSV-treated mice or sera from hRSV-infected mice and then challenged with hRSV, showed reduced pathological signs as compared to nt-hRSV mice (Figure 6C). When mice transferred with sera from rBCG-N+hRSV-treated mice were evaluated, signs of improvement in the lung structure and a decrease in the cellular infiltration were observed as compared to nt-hRSV, being similar to the infiltration found in the lungs from mock-treated mice (Figure 6C).

Equivalent parameters were evaluated for animals transferred with sera from rBCG-P+hMPV-treated mice. As seen in mock-treated mice, we observed almost no hMPV replication and significantly lower viral loads in mice that received sera from rBCG-P+hMPV-treated mice, as compared to non-transferred but hMPV-infected (nt-hMPV), mice transferred with sera from hMPV-infected mice, and from BCG-WT+hMPV-treated mice (Figure 6D). Further, animals transferred with BCG-WT+hMPV-treated mice also showed a decrease in viral loads relative to nt-hMPV, although this was not seen against hMPV-transferred + hMPV mice. Neutrophil infiltration into the lungs was also evaluated for the rBCG-P vaccine. Importantly, sera transfer from rBCG-P+hMPV-treated mice led to a reduced infiltration of neutrophils into lungs, similar to the values seen in mock-treated mice. Further, nt-hMPV mice, mice transferred with sera from hMPV-treated and BCG-WT+hMPV-treated mice showed higher values of neutrophil infiltration, as compared to mice receiving sera from rBCG-P+hMPV-treated mice (Figure 6E). Although animals transferred with sera from BCG-WT+hMPV-treated mice also showed a slight reduction in neutrophil infiltration as compared with the sera from nt-hMPV mice, the value of neutrophils was significantly higher than in those transferred with sera from rBCG-P+hMPV transferred mice (Figure 6E). No significant differences were detected between the hMPV-transferred group and nt-hMPV mice. These data were consistent with the observation that mice receiving sera from rBCG-P+hMPV mice showed a healthier lung structure, similar to the control mock-treated mice, with signs of significantly lower inflammatory cell infiltration, as seen in the histopathological lung samples (Figure 6F). In contrast, mice from groups nt-hMPV, hMPV-transferred, and BCG-WT+hMPV transferred showed a significant loss of lung structure and an increase of inflammatory cell infiltration (Figure 6F). These data suggest that passive transfer of humoral immunity from rBCG-vaccinated mice into *naïve* animals is able to significantly reduce virus-associated pathology symptoms, characterized by viral loads, neutrophil infiltration, and lung structure damage.

## Discussion

Both hRSV and hMPV are respiratory viruses identified as the leading cause of most pathologies affecting the upper and lower respiratory tract of infants, children, elderly and immunocompromised people (10). Both hRSV and hMPV cause bronchiolitis, bronchitis, pneumonia, and high rates of hospitalizations, with hMPV as an emergent pathogen (8, 10–15). Nowadays, no vaccines against these viral pathogens are available. Thus, we developed two vaccines prototype using recombinant BCG strains (rBCG) that incorporate the genes codifying either the N protein (rBCG-N) of hRSV or the P protein (rBCG-P) of hMPV, respectively (24, 25). In this present work, we sought to evaluate whether the previously found cell-mediated immune response protection against hRSV and hMPV was also accompanied by a virus-specific neutralizing antibodies production after viral infection.

Both rBCG-N and rBCG-P have been previously reported as protectors from lung damage, neutrophils infiltration to the lungs and viral replication. Further, these rBCG vaccines were shown to elicit a Th-1 response and induce the proliferation of specific CD4^+^ and CD8^+^ IFNγ^+^ producers T cells (24, 25, 34). Also, rBCG-N has been found to induce a Th-17 response and a long-lasting immunity in mice (34). In the present work, we observed that rBCG-N and rBCG-P were able to induce high antibody titers specific for hRSV and hMPV, respectively, as well as for several of the viral proteins. Interestingly, antibodies against hRSV were found before viral challenge in sera from rBCG-N immunized mice, but no antibodies against hMPV were found in sera from rBCG-P immunized mice before the viral challenge. Further, titers of anti-hMPV, anti-P, anti-M2, and anti-M antibodies were found at 28 dpi in the sera of rBCG-P immunized and hMPV-infected mice. Consistently, it has been previously reported that hMPV displays a biphasic replication cycle and infection peaks fluctuating from 7 to 14 dpi and it is possible to find neutralizing antibodies throughout 28 dpi and until 60 dpi (30). The different behaviors seen between hRSV and hMPV could be associated with the capacity of the N protein of hRSV to migrate to the surface of the host cells, a characteristic that could also enhance its capacity to be presented as an antigen by immune cells (20), when compared with the P protein of hMPV (35). Another reason that could explain the antibody secretion differences found could be associated with the capacity of the BCG to achieve a proper folding of these proteins.

Mature B cells are activated upon B cell receptor (BCR) binding to antigen, which is processed and presented to effector helper T cells (36). Activated B cells will produce antibodies against this antigen. Significantly, even when the concentration and affinity of the antigen is very low, it has been reported that insoluble antigens may congregate in a region of the immunological synapse along with cytoplasmic effectors polarizing inside the B cell, which enhance antigen processing and presentation to T cells (36). We observed that both vaccines induced significant antibody secretion against viral proteins different than those expressed by the rBCGs. Remarkably, the secretion of antibodies against hRSV was significantly higher in the immunized animals (BCG-WT + hRSV and rBCG-N + hRSV), as compared to the unimmunized and infected mice. It has been reported that both hRSV and hMPV infections do not induce an effective humoral immune response, promoting a low antibody secretion with a non-protective isotype (37). On the contrary, we found that BCG-WT, rBCG-N, and rBCG-P promote an increase in antibody secretion throughout time. Such antibody secretion could be explained by the immunogenic capacity of BCG to induce a strong Th-1 profile, prompting the secretion of IFN-γ in higher levels than IL-4 (38). Such Th-1 driving, in turn, promotes the proliferation and differentiation of the B cells population into an effector plasma cell profile, which increase the secretion of antibodies (39, 40). In addition, BCG vaccination by itself has been associated to the selection and survival of B cells and their subsequent maturation toward plasma cell and memory B cell by Follicular B-helper T cell (TFH), which could explain the high antibody titers detected for the mice immunized with the BCG-WT (41). Furthermore, it has been previously described that the immunization with BCG is able to induce the secretion of IgG2a antibodies by itself, therefore explaining the isotope switching detected. Also, the antibodies increase found in rBCG-N when compared to the BCG-WT could be associated with the nature of the antigen and the capacity of the host to present this antigen (39) that could, in turn, promote the enhancement of these capacities of the rBCG, when compared to the BCG-WT strain. Also, it is possible for B cells to be activated recognizing the specific recombinant antigen in their B cell receptor (BCR), while presenting another viral antigen in their Major Histocompatibility complex II (MHC-II) to T cells, for their later activation. These activated T cells will aid naïve B cells to maturate and differentiate into plasma cells, that will secrete high levels of antibodies, as compared with the BCG-WT- which did not express the recombinant antigen- after the viral infection (36). Higher antibody concentrations in sera from hMPV- compared to hRSV-infected mice were found. Such differences could be attributed to the times of collected samples, as the hMPV sera were collected at 28 dpi and the hRSV sera were collected at 14 dpi (40).

Production of IgG specific for an antigen different than the one used for the immunization has been previously reported for other pathogens (42–44). Such phenomenon, known as “linked recognition,” takes place when two or more proteins have epitopes spatially close to each other in a manner that the B cell presents on MHC-II one of those linked antigens to the T cell. As a result, the B cell that receives help from the T cell becomes activated and reacts to the secondary antigen—not the one presented to the T cell—and generates antibodies against this linked antigen. Therefore, it is likely that linked recognition takes place after rBCG-N and rBCG-P immunization, as IgGs specific for other antigens, such as P, F, and G proteins for hRSV and M and M2.1 for hMPV, were produced. These proteins are all closely expressed on the surface of infected cells, along with the N protein during early stages of the replication cycle of hRSV (20). Importantly, the anti-F antibody secretion at 14 dpi from BCG-WT+hRSV and rBCG-N+hRSV mice is significantly higher (about 1,500–2,000 μg/mL) than the one seen for all the other proteins (about 500 μg/mL). Such large antibody titers could be explained by the capacity of both BCG and F protein to activate the TLR4 signaling pathway, which eventually can induce IL-6 secretion (31, 45) and promote secretion of protein specific IgGs by B cells (46).

To corroborate that the linked recognition antibody secretion against the different viral proteins were an effect of the vaccine as a whole and not just an effect of the expressed proteins, we compared the response induced by the transfer of T cells purified from rBCG-N immunized, BCG-WT immunized, and hRSV-infected mice 7 dpi, the point when we detect an early IgG secretion. We also included other groups such as N-hRSV + Freund Adjuvant –as a Th-1 inducer adjuvant- and N-hRSV + Aluminum hydroxide (Alum) Adjuvant –as a Th-2 inducer adjuvant (Supplementary Figure 2). The data obtained indicates that rBCG-N vaccine promotes the highest antibodies secretion when compared with all the other groups, even against N-hRSV protein + Freund Adjuvant –whose adjuvant formulation is based on an extract of BCG.

These results suggest that the live-attenuated vaccine as a whole is required to induce an increase in the humoral immune response. Remarkably, N-hRSV + Alum Adjuvant showed higher levels of antibodies secretion when compared to the other control groups in three of the four analyses made (Supplementary Figure 3). A possible reason for this could be associated with the capacity of this adjuvant to promote a Th-2 immune profile, through the IL-4 secreted by the monocytes –instead of IFN-γ- promoting and strong but not protective humoral response (47). Moreover, it has been reported that the effect of Alum is dependent of several variables, such as the adsorption capacity of the antigen and the protein content of the vaccine, among others (48). Although the IgG measurement was performed at both 7 and 14 dpi, we decided to perform the linked recognition assay at 7 dpi, as we detected that as early as this time point the CD4^+^ T cell population was already activated, therefore the mechanism we suggest could occur at this time (Supplementary Figure 4).

A signature immune response against respiratory viruses, such as hRSV and hMPV, is the antibody secretion of IgG1 and IgG3 isotypes (49, 50). Importantly, although these subclasses can opsonize and neutralize these viruses, they are not suitable for the induction of an effective antiviral response, thereby not being optimal for the control of the infection. Importantly, several studies have demonstrated that IgG2a is the adequate isotype against these infections since it increases the opsonization and exhibits enhanced neutralizing capacities when compared to the IgG1 isotype (51). Interestingly, we found that the use of rBCG strains promotes an isotype switching from IgG1 to IgG2a, which has been previously reported to be associated with an efficient immune response due to the neutralizing activity of these antibodies and the activation of the complement pathway (4). Such isotype changes –along with the Th-1 driving- have already been reported after rBCG immunization (1, 24, 25, 34, 52). Interestingly, we found that rBCG-N vaccine enhanced isotype switching, prompting to an even better humoral response. Also, rBCG-P and BCG-WT promoted the isotype switching seen in rBCG-N—for hMPV-infected mice—but in lower levels when compared with rBCG-N. Such a difference among viruses could be explained because IgG2a displays a faster viral clearance as compared to IgG1, thereby allowing rapid elimination of the pathogen (40). Further, the differences could also be associated to the fact that the IgG2a/IgG1 ratio was determined for the sera obtained at 14 dpi for hRSV and at 28 dpi for hMPV, promoting in this way the decrease of the total circulating IgG.

The use of IFN-α previous to an hRSV-infection has been associated with an increase in the IgA secretion in neonatal and adult mice, accompanied by a strong B cell activation and maturation (53). This effect has also been observed after the BCG vaccination where, using blood samples from neonatal vaccinated children, it was found that BCG promotes the secretion of type I IFN by the plasmacytoid dendritic cells. This might explain why BCG is a good vaccine against viral and intracellular pathogens as we have found in our study (54). The neonatal immune response induced by hRSV-infection promotes an inefficient antibody secretion during the first infection, as compared with adult mice (55). Such a response is characterized with low levels of IgM and IgG in the sera, with a peak at day 28 post-infection, instead of the 7-day post-infection peak detected in adult mice (55). A possible explanation for this behavior is the associated age response dependent-limitation. Also, the antibody levels increase faster after a second infection (55). Therefore, there seem to be differences in the immune response of neonatal compared to adult mice, which could vary the response to the vaccination. For this reason, further studies in neonatal mice are needed to better understand the response of vaccines against hRSV to be intended finally to vaccinate newborns.

As we determined that both rBCG were able to induce the secretion of several types of antibodies, we then sought to evaluate their neutralizing capacities. We found that sera from rBCG-N+hRSV and BCG-WT+hRSV mice at 14 dpi were able to neutralize hRSV-GFP in Hep-2 cell culture since the hRSV PFUs in these cells significantly decreased, shown as an increase in the percentage of inhibition. Importantly, such inhibition was also found in BCG-WT+hRSV sera but its effect was not as high as the one seen in rBCG-N+hRSV sera, suggesting that the BCG could promote the isotype antibodies switching, inducing an inhibitory effect on the capacity of hRSV to infect cells *ex vivo*. Likewise, rBCG-P+hMPV and BCG-WT+hMPV displayed higher percentage of inhibition when compared with mock-treated sera. The hMPV sera also presented significantly higher capacities to neutralize hMPV-GFP in cell culture. These data suggest that a second challenge with hMPV could induce a positive effect on the neutralizing capacity of IgG2a antibodies promoting the control of the viral replication in both the infection alone and the rBCG-P vaccine. Also, although the neutralizing levels in those three groups—hMPV, BCG-WT+hMPV, and rBCG-P+hMPV—were similar, the quantity of secreted antibodies was significantly different among them, suggesting that the vaccine not only promoted the isotype switching, but also a higher secretion of these antibodies.

Given that both vaccines showed neutralizing capacity of the secreted antibodies *ex vivo*, we used these sera to perform a passive transference of immunity to *naïve* mice. In the sera transfer experiments, sera from hRSV-infected mice resulted in a mildly decrease of neutrophils infiltration to the lungs, but only sera from rBCG-N and hRSV-infected mice induce a reduction of neutrophils to mock-like levels. Remarkably, we observed that sera from previously rBCG-N and rBCG- P immunized mice were able to reduce viral load, neutrophils infiltration to lungs and protected from lung damage when compared to their respective control groups.

Currently, the most effective FDA approved treatment against hRSV is palivizumab, a monoclonal antibody that targets a region of F protein that is highly conserved between both antigenic groups of the virus. Further, such antibody is currently used as a prophylactic method, mainly for high-risk infants (51). However, a major concern about this treatment is its price/effectiveness relation, since at least five doses are required in order to achieve a passive immune response. Moreover, even after the administration of five doses, only a 50% of the cases reported a decrease in the hRSV-associated disease parameters. Accordingly, no memory immune response has been reported after administration of palivizumab (19, 51, 56). Importantly, in the sera transfer experiment we found that our rBCG-N strain is able to induce an even more pronounced humoral-mediated protection than palivizumab. Such protection could be associated with the high levels of anti-viral antibodies in the sera of immunized animals and the significant isotype switching, which in turn promote an effective antiviral response, as well as the activation of complement pathway (19).

In conclusion, the use of BCG as a vector could be considered as a promising vaccine approach against respiratory viruses, promoting an efficient humoral response characterized by high titers of neutralizing and protecting antibodies. Thus, our rBCG strains are not inducing only a cellular response, as previously described, but also a humoral response, mediated by neutralizing antibodies against several viral proteins, that promotes an effective immune response when those are transferred to *naïve* recipient mice.

## Author Contributions

JS and NG are equal contributors in the experimental development and design, data organization, data analysis, writing, and revision of this manuscript. CR, CP, and PC supported in organization and experimental design, along with manuscript writing, and revision. ER-J and SB supported in experimental design along with manuscript revision. AK is the leading investigator and supported in the organization, experimental design, and full manuscript revision.

### Conflict of Interest Statement

A patent for the rBCG-N-hRSV has been filled and issued in several countries. The authors declare that the research was conducted in the absence of any commercial or financial relationships that could be construed as a potential conflict of interest

## References

[1] Zhang G Wang P Qiu Z Qin X Lin X Li N. Distant lymph nodes serve as pools of Th1 cells induced by neonatal BCG vaccination for the prevention of asthma in mice. Allergy Eur J Allergy Clin Immunol. (2013) 68:330–8. 10.1111/all.12099 23346957

[2] Dennehy M Bourn W Steele D Williamson AL. Evaluation of recombinant BCG expressing rotavirus VP6 as an anti-rotavirus vaccine. Vaccine (2007) 25:3646–57. 10.1016/j.vaccine.2007.01.087 17339069

[3] Fennelly GJ Flynn JL Ter Meulen V Liebert UG Bloom BR. Recombinant bacille calmette-guérin priming against measles. J Infect Dis. (1995) 172:698–705. 10.1093/infdis/172.3.698 7658061

[4] Matsumoto S Yukitake H Kanbara H Yamada H Kitamura A Yamada T. *Mycobacterium* bovis bacillus calmette-guerin induces protective immunity against infection by *Plasmodium yoelii* at blood-stage depending on shifting immunity toward Th1 type and inducing protective IgG2a after the parasite infection. Vaccine (2000) 19:779–87. 10.1016/S0264-410X(00)00257-7 11115699

[5] Rezende CAF De Moraes MTB De Souza Matos DC Mcintoch D Armoa GRG. Humoral response and genetic stability of recombinant BCG expressing hepatitis B surface antigens. J Virol Methods (2005) 125:1–9. 10.1016/j.jviromet.2004.11.026 15737410

[6] Medeiros MA Armôa GRG Dellagostin OA McIntosh D. Induction of humoral immunity in response to immunization with recombinant *Mycobacterium bovis* BCG expressing the S1 subunit of *Bordetella pertussis* toxin. Can J Microbiol. (2005) 51:1015–20. 10.1139/w05-095 16462859

[7] Wang H Liu Q Liu K Zhong W Gao S Jiang L. Immune response induced by recombinant *Mycobacterium bovis* BCG expressing ROP2 gene of *Toxoplasma gondii*. Parasitol Int. (2007) 56:263–8. 10.1016/j.parint.2007.04.003 17587637

[8] Nair H Nokes DJ Gessner BD Dherani M Madhi SA Singleton RJ. Global burden of acute lower respiratory infections due to respiratory syncytial virus in young children: a systematic review and meta-analysis. Lancet (2010) 375:1545–55. 10.1016/S0140-6736(10)60206-1 20399493 PMC2864404

[9] Lefebvre A Manoha C Bour J-B Abbas R Fournel I Tiv M. Human metapneumovirus in patients hospitalized with acute respiratory infections: a meta-analysis. J Clin Virol. (2016) 81:68–77. 10.1016/j.jcv.2016.05.015 27337518 PMC7106388

[10] Berry M Gamieldien J Fielding B. Identification of new respiratory viruses in the new millennium. Viruses (2015) 7:996–1019. 10.3390/v7030996 25757061 PMC4379558

[11] Edwards KM Zhu Y Griffin MR Weinberg GA Hall CB Szilagyi PG. Burden of human metapneumovirus infection in young children. N Engl J Med. (2013) 368:633–43. 10.1056/NEJMoa1204630 23406028 PMC3662802

[12] Williams JV Harris PA Tollefson SJ Halburnt-Rush LL Pingsterhaus JM Edwards KM Wright PF Crowe JE. Human metapneumovirus and lower respiratory tract disease in otherwise healthy infants and children. N Engl J Med. (2004) 350:443–50. 10.1056/NEJMoa025472 14749452 PMC1831873

[13] Tregoning JS Schwarze J. Respiratory viral infections in infants: causes, clinical symptoms, virology, and immunology. Clin Microbiol Rev. (2010) 23:74–98. 10.1128/CMR.00032-09 20065326 PMC2806659

[14] Hahn A Wang W Jaggi P Dvorchik I Ramilo O Koranyi K. Human metapneumovirus infections are associated with severe morbidity in hospitalized children of all ages. Epidemiol Infect. (2013) 141:2213–23. 10.1017/S0950268812002920 23290557 PMC4106043

[15] Afonso CL Amarasinghe GK Bányai K Bào Y Basler CF Bavari S. Taxonomy of the order Mononegavirales: update 2016. Arch Virol. (2016) 161:2351–60. 10.1007/s00705-016-2880-1 27216929 PMC4947412

[16] Collins PL Melero JA. Progress in understanding and controlling respiratory syncytial virus: still crazy after all these years. Virus Res. (2011) 162:80–99. 10.1016/j.virusres.2011.09.020 21963675 PMC3221877

[17] Mukherjee S Lukacs NW. Innate immune responses to respiratory syncytial virus infection. Curr Top Microbiol Immunol. (2013) 372:139–54. 10.1007/978-3-642-38919-1_7 24362688

[18] Espinoza JA Bueno SM Riedel CA Kalergis AM. Induction of protective effector immunity to prevent pathogenesis caused by the respiratory syncytial virus. Implications on therapy and vaccine design. Immunology (2014) 143:1–12. 10.1111/imm.12313 24801878 PMC4137950

[19] Gomez RS Guisle-Marsollier I Bohmwald K Bueno SM Kalergis AM. Respiratory syncytial virus: pathology, therapeutic drugs and prophylaxis. Immunol Lett. (2014) 162:237–47. 10.1016/j.imlet.2014.09.006 25268876

[20] Céspedes PF Bueno SM Ramírez BA Gomez RS Riquelme SA Palavecino CE. Surface expression of the hRSV nucleoprotein impairs immunological synapse formation with T cells. Proc Natl Acad Sci USA. (2014) 111:E3214–23. 10.1073/pnas.1400760111 25056968 PMC4128097

[21] Caly L Ghildyal R Jans DA. Respiratory virus modulation of host nucleocytoplasmic transport; target for therapeutic intervention? Front Microbiol. (2015) 6:848. 10.3389/fmicb.2015.00848 26322040 PMC4536372

[22] Derdowski A Peters TR Glover N Qian R Utley TJ Burnett A. Human metapneumovirus nucleoprotein and phosphoprotein interact and provide the minimal requirements for inclusion body formation. J Gen Virol. (2008) 89:2698–708. 10.1099/vir.0.2008/004051-0 18931065 PMC2876975

[23] Goutagny N Jiang Z Tian J Parroche P Schickli J Monks BG. Cell type-specific recognition of human metapneumoviruses (HMPVs) by retinoic acid-inducible gene I (RIG-I) and TLR7 and viral interference of RIG-I ligand recognition by HMPV-B1 phosphoprotein. J Immunol. (2010) 184:1168–79. 10.4049/jimmunol.0902750 20042593 PMC2834787

[24] Bueno SM González PA Cautivo KM Mora JE Leiva ED Tobar HE. Protective T cell immunity against respiratory syncytial virus is efficiently induced by recombinant BCG. Proc Natl Acad Sci USA. (2008) 105:20822–7. 10.1073/pnas.0806244105 19075247 PMC2634951

[25] Palavecino CE Cespedes PF Gomez RS Kalergis AM Bueno SM Céspedes PF. Immunization with a recombinant bacillus calmette-guerin strain confers protective Th1 immunity against the human metapneumovirus. J Immunol. (2014) 192:214–23. 10.4049/jimmunol.1300118 24319265

[26] Espinoza JA Bohmwald K Cespedes PF Gomez RS Riquelme SA Cortes CM. Impaired learning resulting from respiratory syncytial virus infection. Proc Natl Acad Sci USA. (2013) 110:9112–7. 10.1073/pnas.1217508110 23650398 PMC3670318

[27] Reina J Ferres F Alcoceba E Mena A de Gopegui ER Figuerola J. Comparison of different cell lines and incubation times in the isolation by the shell vial culture of human metapneumovirus from pediatric respiratory samples. J Clin Virol. (2007) 40:46–9. 10.1016/j.jcv.2007.06.006 17644404

[28] Tollefson SJ Cox RG Williams JV. Studies of culture conditions and environmental stability of human metapneumovirus. Virus Res. (2010) 151:54–9. 10.1016/j.virusres.2010.03.018 20380856 PMC2894476

[29] Céspedes PF Gonzalez PA Kalergis AM. Human metapneumovirus keeps dendritic cells from priming antigen-specific naive T cells. Immunology (2013) 139:366–76. 10.1111/imm.12083 23374037 PMC3701183

[30] Alvarez R Harrod KS Shieh WJ Zaki S Tripp RA. Human metapneumovirus persists in BALB/c mice despite the presence of neutralizing antibodies. J Virol. (2004) 78:14003–11. 10.1128/JVI.78.24.14003-14011.2004 15564507 PMC533920

[31] Marr N Turvey SE. Role of human TLR4 in respiratory syncytial virus-induced NF-κB activation, viral entry and replication. Innate Immun. (2012) 18:856–65. 10.1177/1753425912444479 22535679

[32] Lindblad EB. Aluminium adjuvants - in retrospect and prospect. Vaccine (2004) 22:3658–68. 10.1016/j.vaccine.2004.03.032 15315845

[33] Egli A Santer DM Barakat K Zand M Levin A Vollmer M. Vaccine adjuvants - understanding molecular mechanisms to improve vaccines. Swiss Med Wkly. (2014) 144:w1394. 10.4414/smw.2014.13940 24844935

[34] Céspedes PF Rey-Jurado E Espinoza JA Rivera CA Canedo-Marroquín G Bueno SM. A single, low dose of a cGMP recombinant BCG vaccine elicits protective T cell immunity against the human respiratory syncytial virus infection and prevents lung pathology in mice. Vaccine (2017) 35:757–66. 10.1016/j.vaccine.2016.12.048 28065474

[35] Oliveira AP Simabuco FM Tamura RE Guerrero MC Ribeiro PGG Libermann TA. Human respiratory syncytial virus N, P, and M protein interactions in HEK-293T cells. Virus Res. (2013) 177:108–12. 10.1016/j.virusres.2013.07.010 23892143

[36] Batista FD Iber D Neuberger MS. B cells acquire antigen from target cells after synapse formation. Nature (2001) 411:489–94. 10.1038/35078099 11373683

[37] Freitas GRO Silva DAO Yokosawa J Paula NT Costa LF Carneiro BM. Antibody response and avidity of respiratory syncytial virus-specific total IgG, IgG1, and IgG3 in young children. J Med Virol. (2011) 83:1826–33. 10.1002/jmv.22134 21837801

[38] Finkelman FD Holmes J Katona IM Urban JF Beckmann MP Park LS. Lymphokine control of *in vivo* immunoglobulin isotype selection. Annu Rev Immunol. (1990) 8:303–33. 10.1146/annurev.iy.08.040190.001511 1693082

[39] Drowart A Selleslaghs J Yernault JC Valcke C De Bruyn J Huygen K. The humoral immune response after BCG vaccination: an immunoblotting study using two purified antigens. Tuber Lung Dis. (1992) 73:137–40. 10.1016/0962-8479(92)90146-B 1421345

[40] Mattes MJ Natale A Goldenberg DM Mattes MJ. Rapid blood clearance of immunoglobulin G2a and immunoglobulin G2b in nude mice. Cancer Res (1991) 51:3102–7. 2039990

[41] Moliva JI Turner J Torrelles JB. Immune responses to bacillus Calmette-Guérin vaccination: why do they fail to protect against *mycobacterium tuberculosis*? Front Immunol. (2017) 8:407. 10.3389/fimmu.2017.00407 28424703 PMC5380737

[42] Davies JD Leong LY Mellor A Cobbold SP Waldmann H. T cell suppression in transplantation tolerance through linked recognition. J Immunol. (1996) 156:3602–7. 8621893

[43] Morse K Norimine J Palmer GH Sutten EL Baszler TV Brown WC. Association and evidence for linked recognition of type IV secretion system proteins VirB9-1, VirB9-2, and VirB10 in Anaplasma marginale. Infect Immun. (2012) 80:215–27. 10.1128/IAI.05798-11 22038917 PMC3255688

[44] Fucs R Jesus JT Souza Junior PH Franco L Vericimo M Bellio M. Frequency of natural regulatory CD4+CD25+ T lymphocytes determines the outcome of tolerance across fully mismatched MHC barrier through linked recognition of self and allogeneic stimuli. J Immunol. (2006) 176:2324–9. 10.4049/jimmunol.176.4.2324 16455989

[45] Begum NA Ishii K Kurita-Taniguchi M Tanabe M Kobayashi M Moriwaki Y. *Mycobacterium bovis* BCG cell wall-specific differentially expressed genes identified by differential display and cDNA subtraction in human macrophages. Infect Immun. (2004) 72:937–48. 10.1128/IAI.72.2.937-948.2004 14742539 PMC321570

[46] Suematsu S Matsuda T Aozasa K Akira S Nakano N Ohno S. IgG1 plasmacytosis in interleukin 6 transgenic mice. Proc Natl Acad Sci USA. (1989) 86:7547–51. 10.1073/pnas.86.19.7547 2798426 PMC298102

[47] Ulanova M Tarkowski A Hahn-Zoric M Hanson LÅ. The common vaccine adjuvant aluminum hydroxide up-regulates accessory properties of human monocytes via an interleukin-4-dependent mechanism. Infect Immun. (2001) 69:1151–9. 10.1128/IAI.69.2.1151-1159.2001 11160013 PMC97997

[48] al-Shakhshir R Regnier F White JL Hem SL. Effect of protein adsorption on the surface charge characteristics of aluminium-containing adjuvants. Vaccine (1994) 12: 472–4. 10.1016/0264-410X(94)90127-9 8023556

[49] Knudson CJ Hartwig SM Meyerholz DK Varga SM. RSV vaccine-enhanced disease is orchestrated by the combined actions of distinct CD4 T cell subsets. PLoS Pathog. (2015) 11:e1004757. 10.1371/journal.ppat.1004757 25769044 PMC4358888

[50] Cseke G Wright DW Tollefson SJ Johnson JE Crowe JE. Human metapneumovirus fusion protein vaccines that are immunogenic and protective in cotton rats. J Virol. (2007) 81:698–707. 10.1128/JVI.00844-06 17050599 PMC1797435

[51] Huang K Incognito L Cheng X Ulbrandt ND Wu H. Respiratory syncytial virus-neutralizing monoclonal antibodies motavizumab and palivizumab inhibit fusion. J Virol. (2010) 84:8132–40. 10.1128/JVI.02699-09 20519399 PMC2916538

[52] Cautivo KM Bueno SM Cortes CM Wozniak A Riedel CA Kalergis AM. Efficient lung recruitment of respiratory syncytial virus-specific Th1 cells induced by recombinant bacillus calmette-guerin promotes virus clearance and protects from infection. J Immunol. (2010) 185:7633–45. 10.4049/jimmunol.0903452 21084664

[53] Hijano DR Siefker DT Shrestha B Jaligama S Vu LD Tillman H. Type I interferon potentiates IgA immunity to respiratory syncytial virus infection during infancy. Sci Rep. (2018) 8: 11034. 10.1038/s41598-018-29456-w 30038294 PMC6056463

[54] Kativhu CL Libraty DH. A model to explain how the bacille calmette guérin (BCG) vaccine drives interleukin-12 production in neonates. PLoS ONE (2016) 11:e0162148. 10.1371/journal.pone.0162148 27571272 PMC5003384

[55] Tasker L Lindsay RWB Clarke BT Cochrane DWR Hou S. Infection of mice with respiratory syncytial virus during neonatal life primes for enhanced antibody and T cell responses on secondary challenge. Clin Exp Immunol. (2008) 153:277–88. 10.1111/j.1365-2249.2008.03591.x 18549446 PMC2492908

[56] Hamelin ME Couture C Sackett M Kiener P Suzich JA Ulbrandt N. The prophylactic administration of a monoclonal antibody against human metapneumovirus attenuates viral disease and airways hyperresponsiveness in mice. Antivir Ther. (2008) 13:39–46. 18389897

