# Supplementary material for: Recombinant BCG Vaccines Reduce Pneumovirus-Caused Airway Pathology by Inducing Protective Humoral Immunity
Source: Front Immunol. 2018 Dec 6;9:2875. doi: 10.3389/fimmu.2018.02875 (PMC6293239; doi:10.3389/fimmu.2018.02875)
Supplement: Supplementary file 1 [file Data_Sheet_1.docx]

Supplementary Material

**Recombinant BCG vaccines reduce pneumovirus-caused airway pathology by inducing protective cellular and humoral immunity**

Jorge A. Soto^+1^, Nicolás M. S. Galvez^+1^, Claudia A. Rivera^1^, Christian E. Palavecino^1^, Pablo F. Céspedes^1^, Emma Rey-Jurado^1^, Susan M. Bueno^1^, Alexis M. Kalergis*^1,2^.

*** Correspondence:** Dr. Alexis M. Kalergis; akalergis@bio.puc.cl


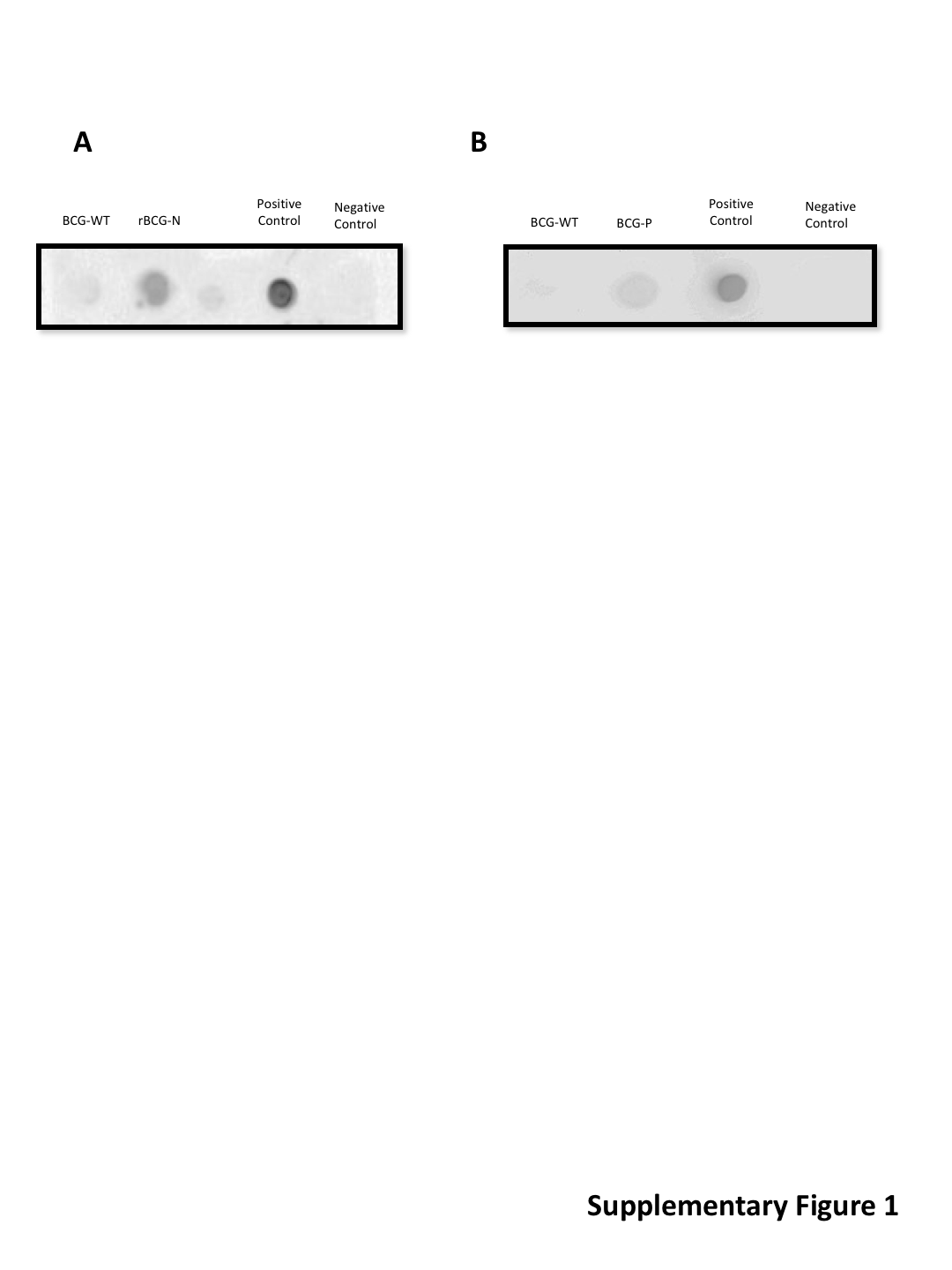


**Supplementary Figure 1. Characterization of recombinant BCGs strains.** The presence of hRSV-N (A) and hMPV-P (B) protein in both recombinant BCG strains were assessed by Dot Blot. Purified N and P protein (25 µg/well) were used as positive control and 1X PBS and BCG-WT were used as respective negative controls.


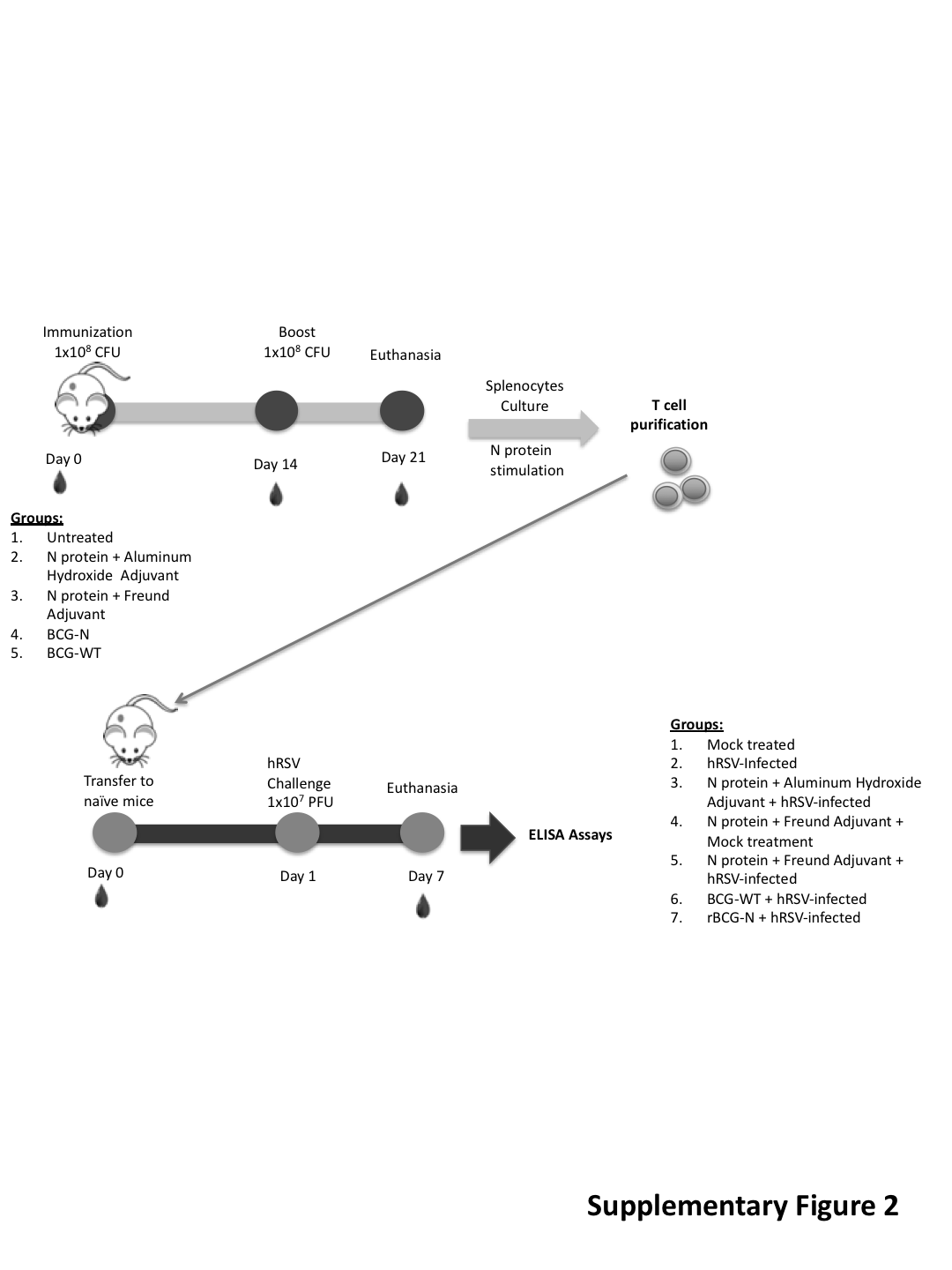


**Supplementary Figure 2. Scheme Linked Recognition.** The experimental design made to confirm the Linked Recognition mechanism was followed such as is showed in the picture. Five experimental groups were treated and 21 days post-treatment the T cell were purified and transferred (Upper panel picture) to seven different experimental groups in naïve mice. These groups were hRSV-infected one day post-transferred and euthanized 7 days post- infection (Lower panel picture).


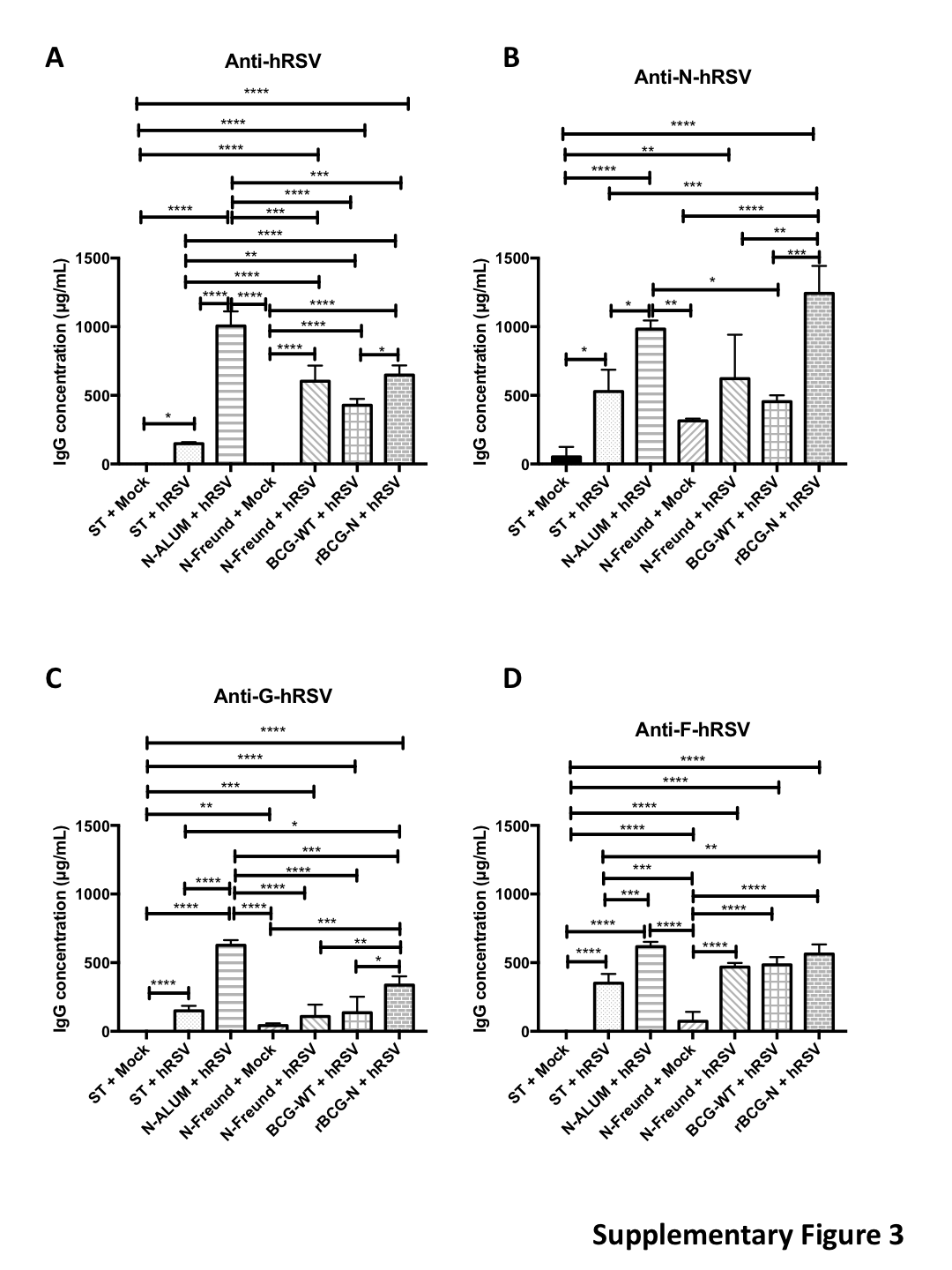


**Supplementary Figure 3**. The rBCG-N strain promotes the Linked Recognition in animals transferred with specific T cells –N hRSV. The secretion of specific antibody against the N- F- G- hRSV proteins and the viral protein extract were measured by indirect ELISA from sera collected of transferred and infected mice after 7 dpi. The anti- hRSV (A), anti-N- hRSV (B), anti-F- hRSV (C) and anti-G- hRSV (D) were evaluated and measured at 450nm. The control groups used in this experiment were N- hRSV protein + Aluminum hydroxide Adjuvant infected, N- hRSV protein + Freund Adjuvant infected, N- hRSV protein + Freund Adjuvant mock-treated. The bars observed in the Figures A-D are accordingly:
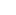
 Mock-treated,
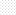
hRSV-infected,
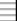
 N protein + Aluminum hydroxide Adjuvant + hRSV,
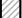
 N protein + Freund Adjuvant + Mock,
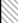
 N protein + Freund Adjuvant + hRSV,
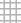
BCG-WT +hRSV and
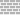
rBCG-N +hRSV. Differences were evaluated by a one-way ANOVA (*=p<0,05; **=p<0,01; ***=p≤0,001; ****=p≤0,0001).


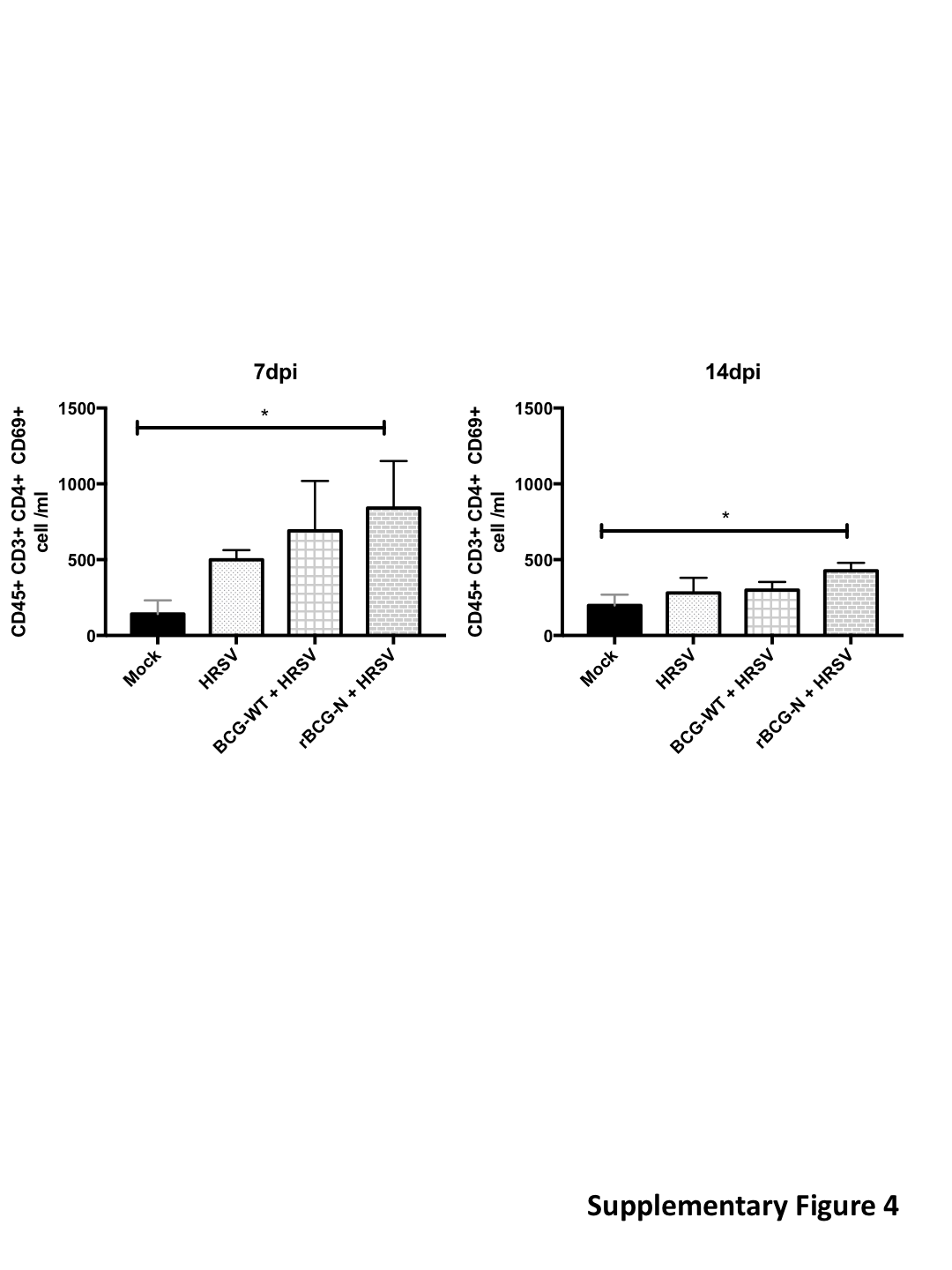


**Supplementary Figure 4**. CD4+ CD69+ T cell populations after 7 and 14 dpi. Mice were immunized with the respective bacteria and then infected with hRSV. T cells populations were characterized in order to determine which day will be chosen for the linked recognition assay.
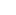
 Mock-treated,
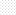
hRSV-infected,
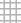
BCG-WT +hRSV and
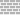
rBCG-N +hRSV. Differences were evaluated by a one-way ANOVA (*=p<0,05; **=p<0,01; ***=p≤0,001; ****=p≤0,0001).
